# Herbal galactagogues to improve breastmilk production and lactation in mothers of preterm babies: a systematic review of clinical trials

**DOI:** 10.1038/s41430-025-01679-x

**Published:** 2025-12-05

**Authors:** Aislinn Cragg, Ilana Levene, Sharram Darabi, Merlin Willcox

**Affiliations:** 1 Primary Care Research Group, Primary Care and Population Sciences, Faculty of Medicine, University of Southampton, Southampton, UK; 2 John Radcliffe Hospital, Oxford, UK

**Keywords:** Nutrition, Paediatrics

## Abstract

Premature infants suffer from conditions such as necrotising enterocolitis and sepsis, whose risk is reduced by breastmilk. Rates of breastfeeding are lower in premature infants compared to term infants. Insufficient breastmilk is the most commonly cited reason for breastfeeding termination. Herbal medicines are commonly used for promoting breastmilk production, but their safety and efficacy are unclear. We wanted to assess whether specific herbal galactagogues can safely and effectively increase lactation in mothers who delivered prematurely. Six databases were searched (Medline, Embase, CINAHL, AMED, COCHRANE library, ProQuest Dissertations and Theses Global) with no language or date restrictions. We included randomised controlled trials (RCTs) of herbal galactagogue use in preterm infant mothers. Ten RCTs were included, each investigating a different galactagogue or mixture. Two scored ‘high’ for risk of bias, the remainder scored ‘some concerns’. There was low certainty evidence of an increase in milk volumes by day 7 of the intervention period with barley malt and lemon balm (mean difference 149 ml, 95% CI: 38–260); *silymarin* in combination with phosphatidylserine and Galega (mean difference 105 ml, 95% CI: 27–183); *Pimpinella anisum* seed tea (mean difference 98 ml, 95% CI: 63–133); and *Latuca sativa* (lettuce) syrup (mean difference 82 ml, 95% CI: 60–105). There is a lack of high-quality RCTs on herbal galactagogues within the preterm population. There is low certainty evidence that Barley malt with lemon balm, silymarin phytosomes with *Galega, Pimpinella anisum* seed tea, *Moringa oleifera* leaf capsules and *Latuca sativa* (lettuce) syrup increase breastmilk production. Higher-quality trials are needed to confirm this effect.

## Introduction

Annually, approximately 11% of births (14 million babies) are born premature (at less than 37 completed weeks of gestation) [1]. 1 million of these newborns die each year, and it is the second leading cause of death in the under 5 s [2]. The World Health Organisation recommends exclusive breastfeeding for the first 6 months of life [3, 4]. If breastfeeding rates were increased to near universal levels, 12% of deaths in the under 5 s could be averted [5].

Necrotising enterocolitis (NEC) and sepsis are important causes of mortality and morbidity in premature infants; 70% of cases of NEC occur in premature infants [6]. In the US, the incidence of early-onset sepsis in premature infants is 13.5 per 1000 premature births [7], compared to 1 per 1000 of total live births [8]. A 2019 Cochrane review found that donor human milk reduces the risk of NEC compared to infant formula [9]. It is possible that raw maternal milk has an even greater beneficial effect than processed donor milk [10]. Despite this beneficial effect, infants born prematurely are less likely to be breastfed than term infants; in premature infants who are breastfed, the duration of breastfeeding is shorter than in infants born at term [11].

Insufficient breastmilk production is an important cause of breastfeeding cessation. According to the 2017 Scottish Maternal and Infant Nutrition survey [12], 86% of women who stopped breastfeeding reported concerns with milk production. Successfully breastfeeding premature infants presents additional challenges due to immature or absent coordination of swallowing and breathing compared to term babies [13, 14]. This means that many mothers of premature infants in newborn care units need to express their milk [15], often for extended periods of time, which can be difficult [15].

Premature babies are often separated from their mothers in intensive care units, making it more difficult for mothers to maintain their milk supply [16]. Premature babies are more likely to have been born by caesarean section, which also reduces the chance of breastfeeding [17]: in the US the overall caesarean delivery rate was 32.1% in 2022. This fell to 26.3% when only term babies were accounted for. Only 10.38% of babies were born preterm in 2022, but accounted for almost 6% of caesareans [18].

Galactagogues are substances that increase breastmilk production [19, 20]. The two most commonly used pharmacological agents, metoclopramide and domperidone, are both used off-label to increase lactation [21, 22]. The evidence regarding their efficacy has been mixed [23, 24]. A meta-analysis on metoclopramide found that despite significantly increasing serum prolactin, there was no increase in milk production compared to placebo [23]. All included studies gave the intervention group metoclopramide 10 mg TDS except one which gave 10 mg BD. In contrast, the most recent systematic review on domperidone showed the drug increased daily milk volumes after 14 days by 88.3 ml/day (95% CI: 56.8–119.8) compared to placebo [24]. The most significant barrier to the use of these agents in clinical practice is concern about side effects. For example, domperidone is not available in the USA due to case reports of patients receiving domperidone suffering from cardiac arrhythmias, cardiac arrests and sudden death [25].

Herbal galactagogues have been used for thousands of years and remain a popular choice. As early as the first century AD, Dioscorides wrote that both fennel and anise ‘draw down the milk’ [26]. In modern times, inquiries regarding herbal galactagogues at an Australian medicine information centre rose from 0 in 2001 to 23% of calls concerning galactagogue use in 2014 [27]. In a US survey of over 1200 breastfeeding women, 27.7% reported using herbal galactagogues. A survey of 82 US healthcare workers found that 70.4% recommended galactagogues; with fenugreek recommended most commonly [28].

However, the safety of using herbal alternatives while breastfeeding remains largely unknown [29]. The Academy of Breastfeeding Medicine has stated that while herbs have historically been used, which is reassuring, there is little scientific evidence regarding their safety and efficacy. Caution should be exercised around their use due to lack of standardised dosing outside of trial settings and the potential for contaminants in preparations [30].

Preclinical studies have shown that herbs may act through several mechanisms. A hydroalcoholic extract of fennel fruits was able to increase prolactin levels in mice [31]. Isoflavones from chickpea sprouts have an oestrogenic effect in ovariectomized rats by increasing levels of FSH and LH and increasing uterine weight [32]. A scoping review looking at 80 studies found that multiple polyphenols may affect milk production in humans, but currently there is a profound lack of information regarding the mechanisms of action of herbal galactagogues in the existing literature [33].

A number of systematic reviews have previously been carried out looking at herbal galactagogues, with variable findings. We identified one review looking solely at mothers of preterm babies and this investigated non-herbal galactagogues [24] (a previous review by Donovan and Buchanan [34] included two studies, which were included in the aforementioned review; as such, we have not included this study). We identified a further study looking at non-herbal galactagogues in preterm infants, but term infants were also included in this study population [35]. There have been five previous systematic reviews including only herbal galactagogues [36–40], two included only term infants [36, 40], one did not specify the study population [37], and the remaining two included both term and preterm infants [38, 39]. We also identified two reviews that included herbal and non-herbal galactagogues [20, 41], but only one included preterm infants [41].

All these reviews illustrated that herbal galactagogues were able to have some positive effect on breastmilk volumes (Supplementary Table 1).

This review aims to answer whether there are specific herbal galactagogues that can safely and effectively increase lactation in mothers of preterm babies.

## Methods

The review protocol was registered on PROSPERO (CRD42023476811). Electronic searches were conducted in Medline, Embase, CINAHL, AMED, COCHRANE library and ProQuest dissertations and theses global from inception to August 2024. There were no language or date restrictions. We also searched references in relevant studies to identify additional studies. We used search terms for ‘preterm babies’, ‘breastfeeding’, ‘herbal galactogogues’ and ‘randomised controlled trials’ (Supplementary Table 2).

### Inclusion criteria

We included studies in lactating mothers of preterm infants (born at less than 37 weeks of gestational age) who received any herbal medicine to increase milk volume. The primary outcome was increase in breastmilk quantity. Secondary outcomes were safety, adverse effects of treatment and increase in preterm infant weight. Only randomised controlled trials (RCTs) were included. Herbal medicines were compared to placebo, no intervention or a different intervention.

### Study selection

Title, abstracts and full texts were screened in Rayyan [42] by two independent reviewers (AC, SD). Inconsistencies were resolved by discussion involving a third reviewer (MW).

### Data extraction

Data extraction forms were created in Microsoft Excel. One reviewer extracted the data (AC), which was then checked by a second reviewer (MW) to confirm accuracy before analyses. Data extracted included: number and type of participants, details of intervention and control, outcomes measured, and any conflicts of interest/funding source reported by the study authors. We extracted all available data on each outcome: milk volumes for days 1, 4 and 7 of the intervention, total milk volume for intervention duration and infant weights for day 7 of the intervention. When data were not provided in a usable format, we contacted the authors for more information. Where we were unsuccessful in contacting authors, WebPlotDigitizer [43] was used to extract data from published figures (IL).

### Study quality appraisal and risk of bias

Quality of the included RCTs was assessed by two independent reviewers (AC, SD) using the Cochrane Risk of Bias 2 Tool [44].

### Data synthesis

Heterogeneity between study interventions prevented meta-analysis in most cases, therefore we performed a narrative synthesis.

The mean difference between intervention and control groups was used to measure effect size for each outcome, for studies which provided sufficient data. Publication bias was assessed using funnel plots where possible. Statistical analysis used Stata SE v18.0 [45].

When results were reported in grams or ounces rather than millilitres/cubic centimetres, we converted this to millilitres using the density of human milk as 1.03 g/ml [46].

### Certainty of evidence

We assessed the certainty of evidence in the included trials using the GRADE system (IL, MW, AC) [47]. We evaluated methodological limitations (risk of bias), inconsistency, indirectness, imprecision, and publication bias.

## Results

**Fig. 1:**
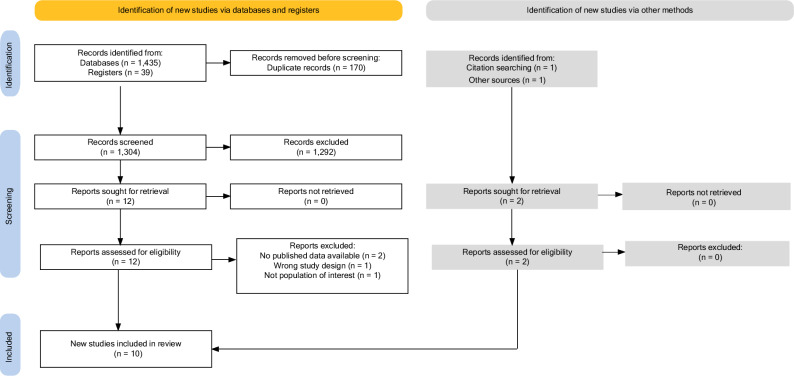
PRISMA flow diagram.

Database searching identified 1474 papers (Fig. 1) [48]. 170 duplicates were removed, leaving 1304 papers for title and abstract screening, of which 12 were selected for full text screening. Citation searching of full texts identified one additional study, and another additional study was provided by an expert. After screening, ten studies, including 856 participants, were eligible for inclusion [49–58].

### Characteristics of included studies

Each study evaluated a different agent, or a mixture of agents (Table 1). Four trials investigated single herbs, as a preparation of the raw herb. Estrella et al. studied *Moringa oleifera* leaf capsules [58]. Reeder et al. investigated fenugreek seed capsules in a small trial of only 26 women [53]. Ranade and Mudgalkar studied *Lepidium sativum* seeds soaked in warm water for 30 min [54], Izaaddoost et al. trialled *Lactuca sativa* (lettuce) syrup [57]. Khalili et al. assessed Anise (*Pimpinella anisum)* seed tea [56].

**Table 1:** Characteristics of included studies.

Author	Country	Participant characteristics	Intervention group^a^	Control/ comparator group^a^	Study duration	Day postpartum intervention initiated	Primary outcomes	Conflicts of interest/funding reported by study authors
Estrella et al. [58]	Philipin es	Mean gestational age of infants (weeks): 33.7 ± 1.9 (intervention) 33.1 ± 2.3 (control) Mean age of mothers (years): 25.8 ± 5.1 (intervention) 30.9 ± 15.7 (control)	*N* = 31 One *Moringa oleifera* leaf capsules containing 250 mg of the leaves—orally twice daily. Total daily dose = 500 mg	Placebo—identical flour capsules (*n* = 37)	5 days (galactagogue and placebo given from day 3 to 5 of study)	Day 3	To determine if there is a significant difference in volume of breastmilk on postpartum days 3 to 5 between those given the placebo and the intervention	Not reported
Reeder et al. [53]	USA	Mean gestational age of infants (weeks): 28.0 (intervention), 28.1 (control) Mean age of mothers (years): 25.8 (intervention) 27.4 (control)	*N* = 14 3 capsules of 575 mg fenugreek orally 3 times daily. Total daily dose = 5175 mg	Placebo—starch capsules (*n* = 12)	21 days	Day 5	Increase in breastmilk volume (no units stated) Increase in prolactin levels in ng/ml Adverse effects	Not reported
Peila et al. [52]	Italy	Mean gestational age of infants (weeks): 29.0 (both intervention and control) Mean age of mothers (years): 35.0 (intervention) 33.0 (control)	*N* = 25 One BIO-C® sachet containing 252 mg micronised silymarin (corresponding to a dose of 420 mg *Silybum marianum)* orally twice daily. Total daily dose = 504 mg of silymarin	Placebo—maltodextrin sachet (*n* = 25)	28 days	Day 10 ± 1	Difference between mean milk production after 26, 27 and 28 days of administration and basal production in g/day	The authors received financial support from Milte Italia S.r.l.—the manufacturer of the herbal product used
Zecca et al. [50]	Italy	Mean gestational age of infants (weeks): 31.5 (intervention) 30.6 (control) Mean age of mothers (years): 34.0 (intervention and control)	*N* = 50 Piùlatte® 5 g sachet containing silymarin- phosphatidylserine (400 mg phytosomes containing 130 mg silymarin and 130 mg phosphatidylserine) with 150 mg galega orally once dailyTotal daily dose = 5 g	Placebo—5 g of lactose (*n* = 25)	25 days	Day 3	Daily volume of milk produced (ml)	The authors declare no conflict of interest
Demirci et al. [49]	USA	Five women delivered between 36 ± 0 and 36 ± 6 weeks, and 6 delivered between 37 ± 0 and 37 ± 6 weeks Participants ranged in age from 23 to 35 years	*N* = 6 Commercially available alcohol-free tincture herbal supplement (Motherlove®: More Milk Plus Alcohol Free containing fenugreek (*Trigonella foenum- graecum*), blessed thistle (*Cnicus benedictus*), nettle (*Urtica dioica*), and fennel (*Foeniculum vulgare*)) Participants were instructed to add the tincture to a palatable liquid <1 oz and drink it 3–4 times per day. The precise dosage was based on the mother’s weight, as advised by the manufacturer.	Meditation intervention via an Apple iPod Shuffle© (*n* = 5) There was a choice of three guided meditations lasting 5–8 min. Participants were instructed to listen to the meditations at least twice each day.	9 days	Days 9–16	Difference between infant weight pre and post intervention (g) Difference between volume of expressed milk pre and post intervention (ml)	The authors declare no conflict of interest
Ozalkaya et al. [51]	Turkey	Mean gestational age of infants (weeks): 30.1 (intervention), 29.1 (control 1), 29.2 (control 2) Mean age of mothers (years): 28.8 (intervention), 29.7 (control 1), 27.7 (control 2)	*N* = 32 8 g orally twice daily of commercially available tea granules (Hipp Natal®) containing 1.0% nettle (*Urtica dioica*) and six other herbs (Melissa (*Melisa officinalis L*.), caraway (*Carum carvi L*.), anise (*Pimpinella anisum*), fennel (*Foeniculum vulgare*), goat’s rue (*Galega officinalis*), and lemon grass (*Cymbopogon citratus*)). Total daily dose = 16 g	Control group 1: placebo—8 g fruit tea (containing hibiscus, rosehip fruit powder, lemon, orange and apple aroma, vitamin C) (*n* = 32) Control group 2: advice on supportive measures (*n* = 21)	7 days	Participants included ranged from a minimum of 3 to a maximum of 47 days postpartum, with a mean of 14–16 days between the three study arms	Daily breastmilk volumes in ml/day Weight gain of preterm infants in g Serum prolactin levels in ng/ml	The authors declare no conflicts of interest.
Ranade and Mudgalkar [54]	India	Mean gestational age of infants (weeks): 31.2 (intervention), 30.2 (control) Mean age of mothers (years): 23.2 (intervention), 24.2 (control)	*N* = 23 2.5 g of *Lepidium sativum* seeds soaked in warm water for 30 min (*n* = 23) orally once daily.	No medication (*n* = 23)	28 days	Not specified	The difference in milk production between the two groups at 28 days (ml) Adverse drug effects	The authors declare no conflict of interest.
Wesolowska et al. [55]	Poland	Mean gestational age of infants (weeks): 31.8 (intervention), 31.4 (control) Mean age of mothers (years): 30.9 (intervention), 31.6 (control)	Femaltiker® sachet (80 mg powdered lemon balm leaves (*Melissa officinalis L*.); 5 g barley malt (*Hordeum vulgare Linn*) enriched with 70% barley beta-glucan—minimum 300 mg beta-glucans from barley per sachet) (*n* = 40) orally twice daily. Total daily dose = 2 sachets	Placebo—a blend of sucrose, apple fibre and natural aroma caramel (*n* = 40)	14 days	Day 2	Total volume of milk expressed by participants from the second to the 14th postnatal day (ml) Safety of the intervention	Nutropharma LLC sponsored the clinical trial—the manufacturer of Femaltiker®. Medela Ltd, Poland, rented the lactation equipment to the study authors free of charge
Khalili et al. [56]	Iran	Mean gestational age of infants (weeks): 30.0(intervention), 30.0 (control 1), 29.0 (control 2) Mean age of mothers (years): 30.7 (intervention), 29.8 (control 1), 32.3 (control 2)	*N* = 45 3 g *Pimpinella anisum* seed herbal tea (2 g of dried anise seeds plus 1 g of black tea) orally 3x daily Total daily dose = 6 g of *Pimpinella anisum* seeds	Control group 1: placebo—3 g of dried black tea (*n* = 45) Control group 2: no treatment (*n* = 39)	7 days	Day 3	Daily milk volume (cc) Infant’s weight at day 3 and day 7 (g)	The authors declare no conflict of interest
Izaddoost et al. [57]	Iran	Gestational age of infants (weeks): 29.91 ± 1.26 (intervention), 29.72 ± 1.25 (control group 1), 29.55 ± 1.03 (control group 2) Mean age of mothers (years): 30.17 ± 5.94 (intervention), 30.43 ± 6.78 (control group 1), 29.66 ± 7.49 (control group 2)	*N* = 50 10 ml orally of *Lactuca sativa* (lettuce) syrup divided 3× daily (syrup standardised based on total phenol content, containing at least 10 mg per 10 g of syrup). Total daily dose = 30 ml	Control group 1: Placebo—sugar syrup (*n* = 50) Control group 2: No intervention (*n* = 50)	8 days	Day 3	Primary outcomes: Time spent pumping milk Frequency of using the pump Time spent skin-to-skin with the infant (kangaroo care), Type and amount of any serums injected into the infant	The authors declare no conflict of interest
							Volume of pumped milk at the end of each day Infant weight at day 1 (before the intervention), day 3, day 5, and day 7 Secondary outcomes: Medication side effects	

^a^We have included the number of participants (indicated by *n*) who completed the study and were therefore not excluded from analysis in this table in the intervention and control groups.

Two trials investigated silymarin, an extract of *Silybum marianum:* Peila et al. [52]. used micronised silymarin (BIO-C®), while Zecca et al. [50]. investigated phytosomes of silymarin with phosphatidylserine and *Galega* (Piùlatte®).

Three commercial combination products were included in the review. Wesolowska et al. trialled ‘Femaltiker®’ (5 g barley malt with 80 mg lemon balm) [55]. Dermirci et al. investigated ‘Motherlove®: More Milk Plus Alcohol Free’, which contained a mixture of fenugreek, nettle, fennel and blessed thistle—as it is a proprietary blend, the proportions of each herb in the mixture were not specified [49]. This study was a very small feasibility trial (9 participants). Ozalkaya et al. investigated a commercially available tea, ‘Hipp Natal®’, containing a proprietary blend of 1.0% stinging nettle and six other herbs (Lemon balm (*Melisa officinalis* L.), caraway (*Carum carvi* L.), anise (*Pimpinella anisum*), fennel (*Foeniculum vulgare*), goat’s rue (*Galega officinalis*), and lemon grass (*Cymbopogon citratus*)) [51].

Most studies used placebo as a comparator [50–53, 55, 56, 58]. Ranade and Mudgalkar compared to no intervention [54], and Demirci et al. compared to meditation, which they considered an active intervention [49]. Three studies had two control groups; in all cases, one was given a placebo. Ozalkaya et al. gave the other advice on supportive measures [51]. Khalili et al. and Izaddoost et al. gave no treatment [56, 57].

Measurement of milk quantity varied slightly between studies. Six of the included studies [50, 51, 53–55, 58] measured milk quantity pumped within a 24-h period using an electric breast pump (the number of times per day women were advised to pump varied between studies from 6 times per day to 8–10 times per day). One study used test weighing of the infant before and after feeds in combination with measuring expressed milk volumes [52]. Two studies [56, 57] used infant weight as an outcome in addition to breastmilk volumes; in one of these studies all infants were weighed at day 0, day 3 and day 7 of the intervention to assess weight gain [56]; in the second study all infants were weighed at day 1, day 3, day 5 and day 7 of the intervention. One study used test weighing or measurement of expressed milk quantity twice a day only, at the time that the mother felt her milk supply was most abundant [49].

### Risk of bias

**Fig. 2:**
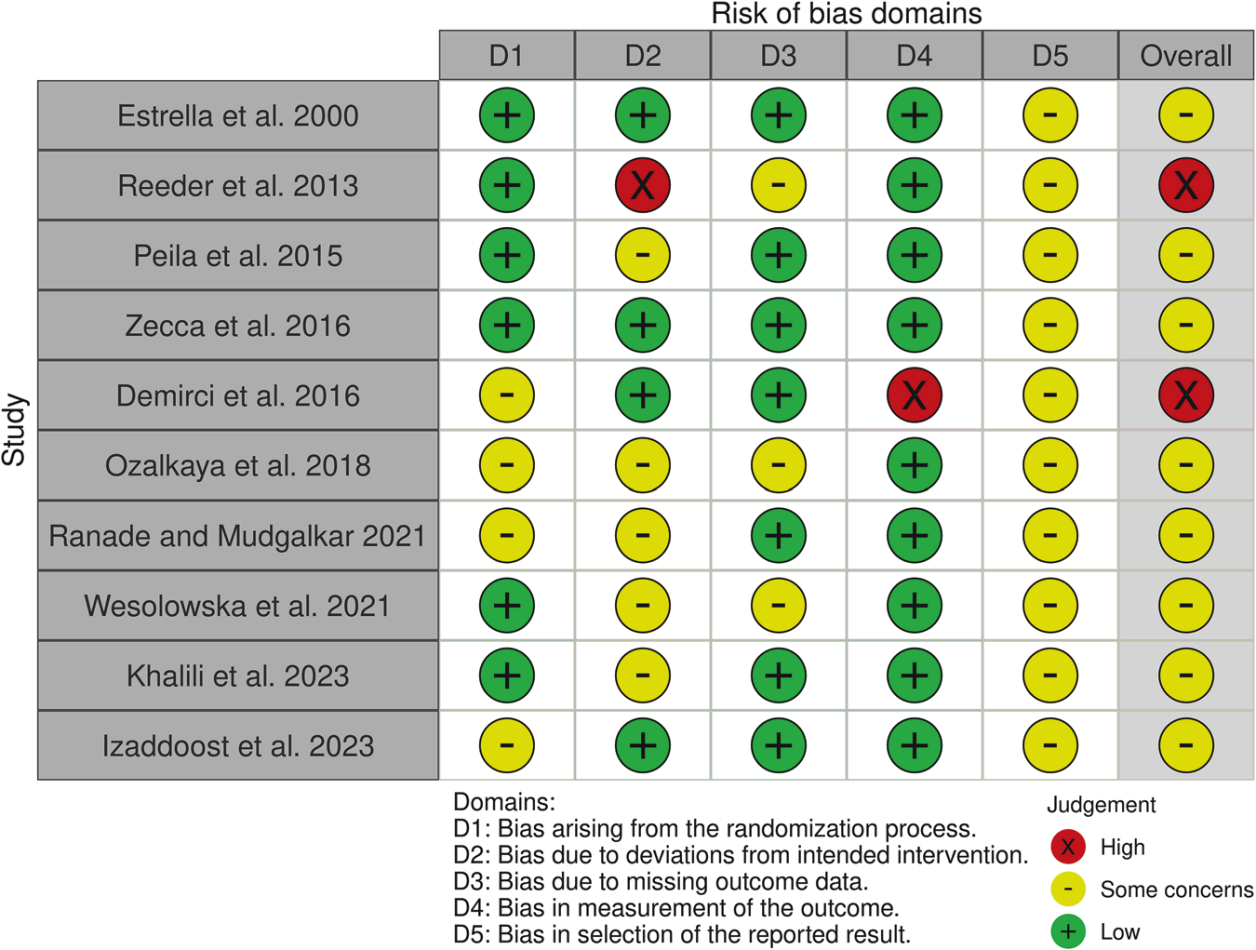
Risk of bias.

Eight of the RCTs had ‘some concerns’ for risk of bias overall [50–52, 54–57], with two assessed as ‘high risk’ [49, 53] (Fig. 2) [59].

Four studies were assessed as ‘some concerns’ for domain one (bias arising from the randomisation process) due to insufficient information about allocation concealment for two studies [51, 54] and imbalance in baseline characteristics for two [49, 57].

Only four studies were assessed as ‘low risk’ for domain two[49, 50, 57, 58] (bias arising due to deviations from the intended intervention); six were assessed as ‘some concerns’ due to a failure to carry out intention-to-treat analysis, resulting in a small number of participants being excluded from data analysis. One was assessed as ‘high risk’ due to a failure to carry out intention-to-treat analysis, resulting in a large number of participant exclusions [53].

Three studies were assessed as ‘some concerns’ for bias in domain 3 (bias due to missing outcome data). One provided no reasons for the loss to follow up of participants [51]. While two provided some reasons for loss to follow-up, there was insufficient detail to be certain that participant drop-out was unrelated to outcome measurement [53, 55]. Reeder et al. [53]. lost over half their participants to follow-up, which was similar between the two study arms.

All studies except one were assessed as ‘low risk’ in domain four (bias in measurement of the outcome), as appropriate methods to measure the outcome were used. The remaining study was assessed as ‘high risk’ for this domain as an inappropriate method, which is prone to bias, was used to measure the desired outcome [49] (mothers pumped milk twice per day at times when milk was perceived to be most abundant).

No study provided a pre-specified statistical analysis plan, so all were assessed as ‘some concerns’ for domain five (bias in selection of the reported result).

There were other limitations of the included studies. One was a feasibility trial with a small sample size [49]. Peila et al. [52]. presented some of their results only as graphs, which could not be extracted with full accuracy. We attempted to contact both study authors but received no reply.

### Synthesis of results

#### Milk volume at day 1 (Supplementary Table 3 and Supplementary Fig. 1)

Four studies provided milk volumes after day 1 of the intervention [54, 56–58]. Mean differences and 95% confidence intervals were calculable for all (Supplementary Table 3 and supplementary Fig. 1). *Moringa oleifera* leaf capsules produced a small increase in milk volume at day 1 compared to placebo, which was not statistically significant (26.9 ml, 95% CI: 22.1–31.6, *P* = 0.052). However, on days 2 and 3, the capsules produced much larger increases in milk volume, which were highly significant (day 2, 66.2 ml, *p* = 0.007; day 3, 199.5 ml, *p* = 0.000) [58]. Two others (*Lepidium sativum* and *Pimpinella anisum*) produced a small increase in milk volume compared to no treatment.

### Milk volume at day 4 (Supplementary Table 4 and Supplementary Fig. 2)

Five studies provided milk volumes for day 4 of the intervention [50, 53, 55–57] ‘Piulatte®’ (silymarin with phosphatidylserine and galega) produced the largest increase (81.8 ml, 95% CI: 17.3–146.4 [50]); followed by ‘Femaltiker®’ (barley malt and lemon balm) (54.1 ml, 95% CI: −16.7 to 124.9, *P* = 0.13 [55]), Anise (44.9 ml, 95% CI: 16.7–73.2, *P* < 0.001 [56]) and *Latuca sativa* (lettuce) syrup (39.2 ml, 95% CI: 22.4–56.0, *P* < 0.001 [57]). In the case of ‘Femaltiker®’ (barley malt and lemon balm), this result was not statistically significant. The study on fenugreek seed capsules [53] (−46 ml, 95% CI: −234 to 142, *P* = 0.645) did not show an increase in milk volume at day 4.

**Fig. 3:**
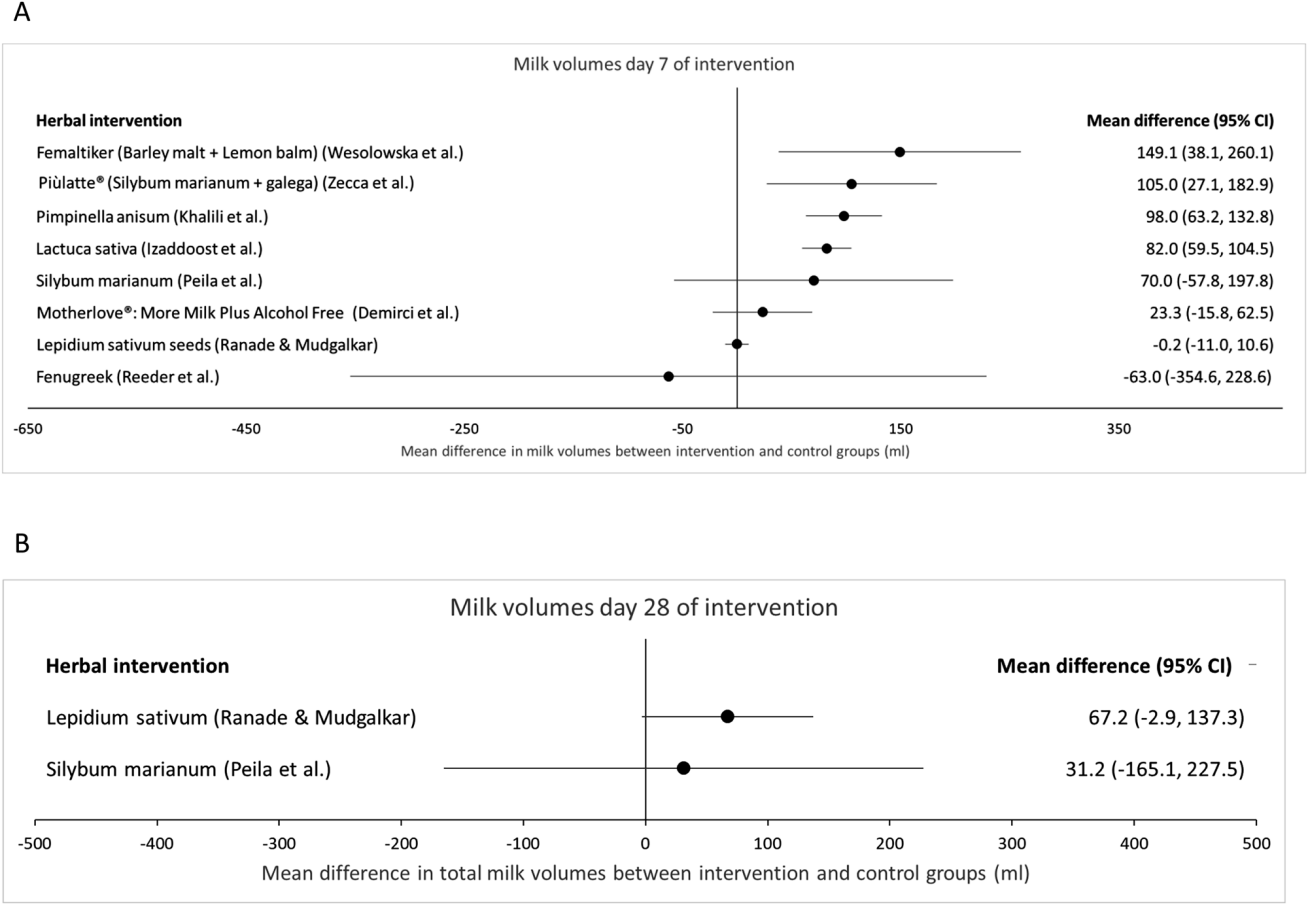
Milk volumes at days 7 and 28 of intervention (ml). **A** Day 7; **B** day 28.

### Milk volume at days 7–10 (Fig. 3a, Supplementary Table 5)

Milk volumes at day 7 or 10 of the intervention period were obtained from nine studies [49–57] (Fig. 3a, Supplementary Table 5). The average participant was between day 9 and day 23 after birth at this time point across the different studies.

There is low certainty evidence that Femaltiker® (barley malt and lemon balm) increases milk volume at day 7 (MD: 149.1 ml, 95% CI: 38.1–260.1; one study, 80 participants) [55]. The certainty of evidence was downgraded by one level for risk of bias and one level for imprecision.

There is low certainty evidence that Piùlatte® (*Silybum marianum* phytosomes with *Galega)* increases milk volume at day 7(MD: 105 ml, 95% CI: 27.1–182.9) [50, 52]. The certainty of evidence was downgraded by one level for risk of bias and one level for imprecision. However, a different preparation of micronised *Silybum marianum* (Bio-C) did not have a significant effect on milk volume at day 7 [52].

There is low certainty evidence that *Pimpinella anisum* tea increases milk volume at day 7 (MD: 98.0 ml, 95% CI: 63.2–132.8; one study, 129 participants) [56]. The certainty of evidence was downgraded by one level for risk of bias and one level for imprecision.

There is low certainty evidence that ‘Hipp Natal®’ granules (containing 1.0% stinging nettle, *Pimpinella anisum* and five other herbs) increased milk volume at day 7 (MD: 99.8 ml, 95% CI: not calculable, *p* = 0.22; one study, 85 participants) [51]. The certainty of evidence was downgraded by one level for risk of bias and one level for imprecision.

There is low certainty evidence that *Latuca sativa* (lettuce) syrup increases milk volume at day 7 (MD: 82.0 ml, 95% CI: 59.5–104.5; one study, 140 participants, *p* < 0.001). The certainty of evidence was downgraded by one level for risk of bias and one level for imprecision.

There is low certainty evidence that *Lepidium sativum* seeds do not change milk volume at day 7 (MD: −0.2 ml, 95% CI: −11.0 to 10.6; one study, 46 participants) [54]. The certainty of evidence was downgraded by one level for risk of bias and one level due to imprecision.

There is very low certainty evidence that ‘Motherlove®: More Milk Plus Alcohol Free’ does not increase milk volume at day 7 (MD: 23.3 ml, 95% CI: −15.8 to 62.5; one study, 9 participants) [49]. The certainty of evidence was downgraded two levels due to risk of bias and one level for imprecision.

There is very low certainty evidence that fenugreek seed capsules do not increase milk volume at day 10 (MD: −63.0 ml, 95% CI: −354.6 to 228.6; one study, 26 participants) [53]. The certainty of evidence was downgraded two levels due to risk of bias and one level for imprecision.

**Fig. 4:**
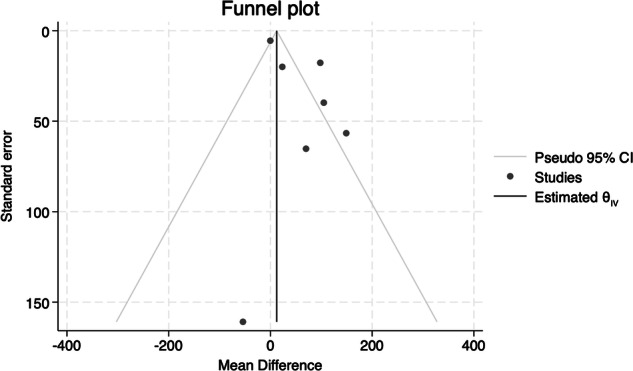
Funnel plot indicating publication bias.

A funnel plot of these studies (Fig. 4) suggests publication bias towards studies with a positive effect (five studies had a positive effect, and only two studies with a negative effect were published).

### Milk volume at day 28 (Fig. 3b, Supplementary Table 6)

There is low certainty evidence that *Lepidium sativum* seeds increase milk volume at day 28 (MD: 67.2 ml, 95% CI: −2.9 to 137.3; one study, 46 participants) [54]. The certainty of evidence was downgraded by one level for risk of bias and one level for imprecision.

There is very low certainty evidence that micronised silymarin does not increase milk volume at day 28 (MD: 31.2 ml, 95% CI: −165.1 to 227.5; one study, 48 participants) [52]. The certainty of evidence was downgraded by one level for risk of bias and two levels for imprecision.

**Fig. 5:**
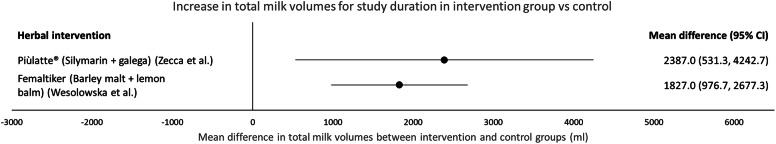
Milk volume over the whole intervention period (ml).

### Milk quantity over the whole intervention period (Fig. 5, Supplementary Table 7)

There is very low certainty evidence that Piulatte® (silymarin phytosomes with phosphatidylserine and galega) increases milk volume throughout the intervention period (25 days) (MD: 2387.0 ml, 95% CI: 531.3–4242.7; one study, 100 participants) [50]. The certainty of evidence was downgraded by one level for risk of bias and two levels for imprecision.

There is very low certainty evidence that Femaltiker® (barley malt and lemon balm) increases milk volume throughout the intervention period (14 days) (MD: 1827 ml, 95% CI: 650.7–3003.4 ml; one study, 80 participants) [55]. The certainty of evidence was downgraded by one level for risk of bias and two levels for imprecision. With Piùlatte®, the increase in milk volume was statistically significant from day 4 onwards [50]. With Femaltiker® (barley malt and lemon balm), milk volumes were measured from days 4 to 11, and the increase in milk volume became statistically significant from day 6 [55].

**Fig. 6:**
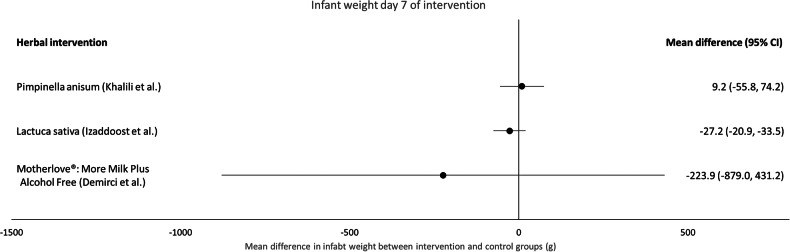
Infant weight at day 7 of intervention (g).

### Infant weight at day 7 of the intervention (Fig. 6, supplementary Table 8)

Four studies [49, 51, 56, 57] reported infant weight as an outcome on day 7 of the intervention (Fig. 6, Supplementary Table 8). In Khalili et al. and Ozalkaya et al.’s studies, infants were also fed formula milk [51, 56], which could confound results.

There is low certainty evidence that *Pimpinella anisum* tea does not increase infant weight (MD: 9.2 g, 95% CI: −55.8 to 74.2; one study, 90 participants) [56]. The certainty of evidence was downgraded by one level for risk of bias and one level for imprecision.

There is low certainty evidence that ‘Hipp Natal®’ granules (containing 1.0% stinging nettle, *Pimpinella anisum* and five other herbs) do not increase infant weight (MD: −3 g, 95% CI: not calculable, *p* = 0.99; one study, 64 participants) [51]. The certainty of evidence was downgraded by one level for risk of bias and one level for imprecision.

There is low certainty evidence that *Latuca sativa* (lettuce) syrup does not increase infant weight (MD: −27.2 g, 95% CI: −74.6 to 20.2, *p* = 0.822; one study, 140 participants). The certainty of evidence was downgraded by one level for risk of bias and one level for imprecision.

There is very low certainty of evidence that ‘Motherlove®: More Milk Plus Alcohol Free’ does not increase infant weight (MD: −223.9 g, 95% CI: −879.0 to 431.2, one study, 9 participants) [49]. The certainty of evidence was downgraded two levels for risk of bias and one level for imprecision.

### Safety of the interventions

Nine studies reported that no participants experienced any side effects; however, only three studies clearly stated how the presence of adverse effects was assessed [51, 54, 55]. Only one study (‘Motherlove®: More Milk Plus Alcohol Free’ [49]) reported a number of minor maternal side effects: headache (*n* = 1), nausea (*n* = 1), bodily smell of maple-syrup (*n* = 2), increased perspiration (*n* = 1). No infant side effects were reported by any included study.

## Discussion

### Summary of findings and comparison with existing literature

Our search identified only 10 eligible RCTs of herbal galactagogues in mothers of preterm babies, all of different products. There were no safety concerns. From the limited available evidence, barley malt with lemon balm powder, silymarin phytosomes with phosphatidylserine and Galega, *Moringa oleifera* leaf capsules, Anise seed tea and *Latuca sativa* (lettuce) syrup appear most promising for further research.

### Barley malt

Barley malt is the major constituent of Femaltiker®, which had a significant effect on breastmilk production from day 6 of intervention [55]. Beer was traditionally consumed to increase lactation; it has been shown that barley malt is the active component [60]. Experiments on ewes found that intravenous administration of barley malt increased blood prolactin levels, suggesting this is the mechanism behind its effect on breastmilk [60]. Barley malt is a safe, inexpensive and widely available plant product, which could be recommended to breastfeeding mothers.

### Silymarin

Silymarin is an extract of the milk thistle plant (*Silybum marianum*), and is believed to be responsible for its therapeutic effects [61]. Silymarin includes four flavonolignans (silybin, silychristin, silydianin and isosilybin), which are phytoestrogens [50]. These may increase breastmilk volumes by acting at D2 receptors to increase prolactin levels [62].

Of the two studies of silymarin, only one showed a significant increase in milk volume after 7 days of using a combination of 400 mg *Silybum marianum* with 130 mg phosphatidylserine, and 150 mg *Galega officinalis* (‘Piulatte®’). The silymarin was combined with the phospholipid in order to create ‘phytosomes’, which are claimed to improve intestinal absorption of the insoluble silymarin.

Although the clinical trial of micronised silymarin (‘BIO-C®’) showed no significant effect [52], a different trial of the same preparation in lactating mothers of term babies [63] (excluded from this review) reported significant increases in milk volume over 63 days compared to placebo (85.9% v 32.1%). The reasons for this different result are not clear, because the same preparation and dose were used; however, the eligibility criteria were not clear in the study of term babies.

### Anise

There was low certainty evidence that Anise *(Pimpinella anisum)* seed tea [56] increased milk volumes in the short term, as the study by Khalili et al. only lasted for 7 days and there was no long-term follow-up of participants.

Hipp Natal® tea was used by Ozalkaya et al. [51]. (which also contained anise) resulted in a statistically significant increase in breastmilk volumes when adjusting for baseline milk volumes (but not at day seven alone as reported above). However, the proportions of the different herbs used in this product were not specified, so the specific role of Anise cannot be deduced from this.

Anise contains anethole, whose structure is similar to dopamine [64]. Dopamine inhibits the release of prolactin. Anethole may compete with dopamine, thus preventing inhibition of prolactin release, allowing prolactin levels to rise [64].

However, both anise and lemon balm contain the aromatic compound estragole [65], which has a dose-dependent genotoxic effect [66]. Animal studies suggested that this effect would likely be minimal in the dose range of 1–10 mg/kg, which is 100–1000 times greater than the expected human exposure to estragole [67]. However, the Herbal Medicinal Products Committee of the European Medicines Agency recently updated its guidance [67], recommending that breastfeeding women should not exceed a daily dose of 0.05 mg of estragole, making regulatory agencies potentially cautious about its use. However, it is unlikely that the dose in a herbal galactagogue preparation would exert a harmful effect. Anise has a long history of use in lactating women, with no known problems, at doses which do not greatly exceed amounts used in foods [68].

### Latuca sativa

*Latuca sativa*, commonly known as lettuce, contains lignan, a component of the phytoestrogen family [69] and flavonoids [70], which may be responsible for its galactagogue effect. Lettuce is widely cultivated [70], making it cheap and readily available. As it is a food, it is very safe for human consumption.

### Moringa oleifera

*M. oleifera*, commonly referred to as the ‘drumstick tree’ because of its large seed pods, is native to the western sub-Himalayan regions of India, Pakistan, Bangladesh and Afghanistan, but has been cultivated for food and medicine in tropical Asia, sub-Saharan Africa, Latin America and the Caribbean [71, 72]. In the Philippines, Moringa is commonly known as ‘Malunggay’, where almost all of the plant is used in multiple industries such as food, cosmetics and herbal medicine [73]. Its leaves, pods and flowers are commonly eaten. The leaves are especially rich in protein, essential amino acids, iron, copper, calcium, Vitamin C and carotenoids [71, 74] and have been promoted as a nutritional supplement for malnourished children and lactating women [75].

Therefore, various non-governmental organisations and governments have supported large-scale planting of *Moringa* [76]. The safety of the leaves has been confirmed by laboratory experiments in rats—even at doses of 2000 mg/kg, no mortality ensued [77].

Moringa has a long history of use in traditional medicine, with both the leaves and seed pods being used [78]. It is believed to increase lactation by increasing prolactin levels; however, its mechanism of action is still unclear [37].

### Fenugreek

There was very low certainty evidence about any effect of fenugreek for mothers of preterm infants in the two included studies, which were both extremely small, so no conclusions can be drawn in this population.

Although fenugreek is commonly used as a galactagogue, its mechanism of action is poorly understood [36]. A 2020 study in rats, which used a dry water extract of fenugreek seeds at 1 g/kg body weight/day, indicated that fenugreek may increase milk production by stimulating insulin secretion and a modulation of the insulin/GH/IGF-1 axis [79].

There has been a previous systematic review of five RCTs of fenugreek as a galactagogue in the mothers of term infants and preterm infants. Aside from the study by Reeder et al, this review included three studies of fenugreek herbal tea and one of fenugreek seed capsules. The review suggested that fenugreek increased breastmilk production more than a placebo but was less effective than two other herbal products, namely *Coleus amboinicus* and palm dates [38].

A number of side effects from fenugreek use have been reported [80]. Fenugreek has the potential to cause anticoagulant effects, and therefore should not be used by women who currently take anticoagulants or women who have a history of clotting disorders [80]. Fenugreek has been associated with nausea, vomiting and diarrhoea [80]. Fenugreek may cause hypotension, so it should be used with caution in those with existing low blood pressure or patients who take anti-hypertensives; however, a previous systematic review concluded it did not cause a significant reduction in systolic or diastolic blood pressure unless doses of 15 g/day or more over a minimum of 12 weeks were used [81]. Therefore, it is unlikely that the dose used to promote lactation would have a harmful effect.

Additionally, fenugreek has been linked to hypoglycaemia [82], and so is not recommended for use in women with insulin-dependent diabetes.

### Study strengths and limitations

The strengths of this review include the following. This review followed the Preferred Reporting Items for Systematic Reviews and Meta-Analyses (PRISMA) guidelines [83]. We conducted a comprehensive search of six databases, including grey literature. The citations of articles meeting our inclusion criteria were also searched. The search strategy used was checked by a librarian with specialist knowledge. We attempted to contact the authors of studies where possible when clarification was required. There were no language or date restrictions. All included studies were double-screened. Data extraction and risk of bias assessments conducted by one reviewer were checked by a second independent reviewer.

Limitations of this review include the following. More articles might have been identified by searching other databases, such as Chinese language databases for herbal medicines. We were only able to identify a small number of studies which met our inclusion criteria, and these studies had a small number of participants. Although we attempted to contact several authors for clarification, not all responded. Meta-analysis was only possible between two studies, which had similar interventions; no other meta-analysis was possible due to heterogeneity between interventions. The funnel plot showed that it is likely that there is publication bias as a whole in the field of herbal galactagogues, and that, therefore, there are probably unpublished studies with negative results.

Our methodology could have been improved by calculating the mean difference in change from baseline between study arms to adjust for differences in milk production at baseline. As not all included studies provided baseline values, this was not possible.

We changed one inclusion criterion from the protocol registered on PROSPERO, which specified we would only include mothers with lactational insufficiency. We broadened this to include all mothers of preterm babies, as there is no clear definition of ‘lactational insufficiency’.

### Implications for policy and practice

All included outcomes were rated as either having low or very low certainty of evidence, so any recommendations must be cautious. Nevertheless, breastfeeding mothers of preterm babies may request advice on the most useful herbal galactagogues and it is important to give evidence-based recommendations. This review suggests that the safest, most effective and affordable ‘natural’ galactagogues for mothers of preterm babies are barley malt, lettuce syrup, and *Moringa oleifera* leaf capsules. *Lepidium sativum* [54] took 28 days to have a significant effect. Despite fenugreek’s common recommendation by healthcare professionals [28]; based on one study in our review, there is insufficient evidence on its efficacy in mothers of preterm babies. There was evidence in favour of silymarin phytosomes (combined with phosphatidylserine) with Galega (Piùlatte® [50]), but as a branded supplement, this is expensive. A box of sachets, which would last for 14 days, currently retails for ~£25 [84], so would not be accessible for all lactating mothers, and would likely not be available in many countries.

### Priorities for future research

Our results suggest that the top priorities are high-quality RCTs of barley malt, *Latuca sativa* (lettuce) syrup, *Moringa oleifera* leaf capsules, and Silymarin phytosomes with Galega in breastfeeding mothers of preterm infants. Given the widespread use of fenugreek in practice, a large RCT would also be useful for this. Trials would also be useful to evaluate the safety and effectiveness of other herbal products which have a significant effect on mothers of term babies, but have not yet been studied in mothers of preterm babies. There is little evidence on the optimal doses required to exert a therapeutic effect for any of these agents, so dose-finding studies [85] are also needed to define the optimal therapeutic doses.

It is important to note that published RCTs used only one form of the aforementioned galactagogues, so we can only draw conclusions based on the effectiveness of those preparations rather than the effectiveness of the herb in general. Different preparations, for example, a capsule containing the dried herb or an aqueous preparation such as a tea of the same herb, may have a different effect. Different parts of the same plant may also produce different results. How each galactagogue would affect milk production in a different form (for example, a tea or tincture), may be a consideration for future research. It is also important to consider that several of our studies used capsules, which is very different to the traditional form in which herbs were traditionally consumed. In future, researchers should consider using herbs in traditional preparations when considering their efficacy. However, this would make blinding difficult, which is why capsules may be more useful when conducting a double-blind trial.

Future RCTs should be correctly powered to allow clinically relevant conclusions to be drawn; double-blinded where possible (although this could be challenging for some products with a distinctive taste or smell); and should provide a pre-specified analysis plan. Confounding factors such as maternal age, milk removal frequency, and delivery method should be recorded and controlled for in the analysis, especially if groups are not well balanced [86]. When using herbal medicines, it is important to ensure product quality and consistency due to factors such as geographical variations in plants and differences in cultivation methods between regions [87].

Our included studies measured outcomes such as breastmilk volumes, infant weight and serum prolactin levels. It could be argued that an increase in breastmilk volume is not an end in itself. For example, the percentage of mothers who continue to breastfeed until their child is 6 months of age is an important outcome, which was not assessed in any included trials. However, the maternal anxiety that surrounds milk volumes following a preterm birth means it is still an important direct outcome to measure.

Four of our included studies measured infant weight as an outcome [49, 51, 56, 57]. Two of these studies [49, 51] stated that infant formula was used as a supplement to maternal milk. In the other two studies [56, 57], it is unclear if infants were given infant formula. The time of day when infants were weighed was only reported by two studies [56, 57]. Standardising when infants are weighed may not be possible in a busy hospital setting. If mothers are responsible for weighing their own infant, this relies on all participants adhering to the study protocol. If milk volumes are insufficient and supplementary infant formula is used, the amount of infant formula used should be measured and included in the analysis. If infant weight is used as an outcome, care should be taken to measure it in such a way that the results are able to provide meaningful information. Seven days is a very short period over which to conduct a study, so future studies should have longer follow-up periods.

More efforts should be made to reduce losses to follow-up by providing incentives for participants to continue, such as offering vouchers, and keeping them informed about the relevance and progress of the study [88].

## Conclusions

Low-certainty evidence suggests that Femaltiker® (barley malt with lemon balm), Anise (*Pimpinella anisum)* seed tea, silymarin phytosomes (with Galega), *Moringa oleifera* leaf capsules and *Latuca sativa* (lettuce) syrup, may be of benefit to lactating mothers of preterm infants. Further high-quality clinical trials are needed for all these, and other herbal galactogogues which are effective in the mothers of term babies. Based on the only currently available study of fenugreek use in this population, there is at present insufficient evidence to recommend fenugreek or other herbal galactogogues for mothers of preterm infants.

## Supplementary information

Supplementary Table 1

Supplementary Table 2

Supplementary Materials 3
