# Supplementary material for: Herbal galactagogues to improve breastmilk production and lactation in mothers of preterm babies: a systematic review of clinical trials
Source: Eur J Clin Nutr. 2025 Dec 5;80(2):146–58. doi: 10.1038/s41430-025-01679-x (PMC12929060; doi:10.1038/s41430-025-01679-x)
Supplement: Supplementary file 1 — Supplementary Table 1 [file 41430_2025_1679_MOESM1_ESM.docx]

| Reference | Number and types of studies included | Population | Interventions | Outcomes reported | Results |
| --- | --- | --- | --- | --- | --- |
| Mortel & Mehta 2013 | 6 RCTs | Term infants | Shatavari *(Asparagus racemosus*) – 2 RCTs  Torbangun (*Coelus amboinicus L*.) – 1 RCT  Milk thistle (*Silybum marianum) –* 1 RCT  Fenugreek (*Trigonella foenum-graecum*) – 1 RCT  Xiong-gui-tiao-xue-yin (Japanese angelica root, *Cnidium* rhizome, *Rehmannia* root, *Atractylodes* rhizome, hoelen, *Citrus unshiu* peel, *Cyperus* rhizome, Moutan bark, Lindera root, jujube fruit, Siberian motherwort herb, ginger rhizome, and *Glycyrrhiza* root) – 1 RCT | Primary outcome:  Breastmilk production  Maternal serum prolactin | Five trials found an increase in breast milk production in those given a galactagogue:  Shatavari:   - 1 RCT showed no statistically significant effect on breastmilk volumes - 2nd RCT showed a statistically significant effect on maternal serum prolactin and infant weight at the end of the 30 day study:   mean percent increase in prolactin level  (SD): 32.9% (6.5) vs 9.6% (4.6) (P < .05);  mean percent increase in infant weight  (SD): 16.1% (3.7) vs 5.7% (2.6) (P < .05)  Milk thistle: RCT found a statistically significant increase in breastmilk production at day 30 of intervention vs placebo: mean breast milk quantity (SD); 990 (102) g vs 650 (78) g (P < .01)  Fenugreek: RCT compared the intervention to a placebo group given apple tea, and a control group given no intervention. The duration of intervention was not reported. By the end of the study those given fenugreek produced significantly more milk than those in the placebo or control groups; mean breast milk volume (SD): 73.2 (53.5) mL; 38.8 (16.3) mL; 31.1 (12.9) mL (P < .05  Torbangun: RCT found that at day 28 of the study mean breast milk volume was significantly increased in the intervention group vs placebo: (SD) 479 (157) mL; vs 385 (202) mL (P < .05)  Xiong-gui-tiao-xue-yin: RCT found enhanced 24-h human milk volume on day 6 postpartum (413.7 ± 68.1 vs. 293.3 ± 98.5 g, p = 0.046) |
| King et al. 2013 | 5 RCTs | Not specified (mothers who took raw or pharmaceutically compounded drug with Moringa oleifera as the active ingredient were included) | Raw or pharmaceutically compounded drug with Malunggay (Moringa oleifera) as the active ingredient | Primary outcomes:  Breastmilk volume  Infant weight gain  Secondary outcomes:  Maternal prolactin levels  Adverse drug reactions | Three of the included studies measured milk volume, two studies measured infant weight, two studies measured maternal prolactin levels. Four trials reported adverse outcomes.  Pooled analysis of the three trials which measured milk volume showed an increase in milk volumes compared to placebo at day 7 (123.87ml [CI 89.48-158.26]), but the result was not statistically significant p = 0.32  Of the two studies which reported on maternal serum prolactin, one found a statistically significant increase in prolactin in those receiving the intervention compared to placebo at 2 days post-partum (median 1884.9 mIU/L [95% CI 1601.8-2168]), and 4 months postpartum (median 1838 mIU/L [95% CI 1073.04-2602.96]). The remaining study found no significant difference between intervention arms  Both studies which reported on infant weight found a % increase in infant weight in the intervention group compared to placebo, but no pooled analysis was available for this outcome.  No adverse effects were reported |
| Bazzano et al. 2016 | 18 RCTs | Term and preterm infants | Domperidone – 4 RCTs  Metoclopramide – 6 RCTs  Fenugreek *(Trigonella foenumgraecum)* – 2 RCTs  Shataviari (*Asparagus racemosus)* – 2 RCTs  Milk thistle (*Silybum marianum)* – 1 RCT  Garlic (*Allium sativum)* – 1 RCT  Malunggay (*Moringa oleifera) –* 2 RCTs | milk production  infant weight gain  maternal serum prolactin level  time to relactation | Domperidone: All included studies of domperidone indicate significantly more milk output in mothers who received domperidone than a placebo (p < 0.05).  Metoclopramide   - Of the 6 metoclopramide studies 5 showed no significant difference in volume of milk production, mean infant weight gain, or time to relactation between those receiving metoclopramide and those receiving placebo. - In one study mean milk production was significantly higher among mothers receiving treatment than among those receiving a placebo (75.4 ± 13.2 g vs 51.1 ± 10.9 g, respectively; P<0.01).   Fenugreek:   - One RCT reported statistically significant increase in breastmilk volumes in those receiving fenugreek (mean milk production 73.2 ± 53.5 mL) compared to placebo (mean milk production 38.8 ± 16.3 mL) (P = 0.004). - The second study reported no significant difference between the intervention and control groups.   Shatavari:   - One RCT found no statistically significant difference between the intervention and placebo groups regarding milk production, infant weight gain or maternal serum prolactin level. - The second RCT found a statistically significant increase in maternal serum prolactin levels in the intervention group compared to placebo (mean prolactin increase of 32.87% ± 6.48% in the treatment group vs a mean increase of 9.56% ± 4.57% in the placebo group frombaseline (P<0.05)). They also reported a statistically significant increase in infant weight (mean increase of the babies’ weight was16.13% ± 3.65% in the treatment group vs 5.68% ± 2.57% in the placebo group (P<0.05)).   Milk thistle: mothers taking silymarin produced significantly (P<0.01) more milk than the mothers taking a placebo (treatment group 989.76 ± 102.33 g, 64.43% increase vs the placebo group 649.76 ± 78.35 g, 22.51% increase).  Garlic: no significant difference was detected between any of the groups at the study’s end.  Malunggay:   - One RCT reported no significant difference in milk volumes between study arms. - One reported statistically significant increases in prolactin levels (5,235 ± 2,252.4 mIU/L vs 3,398 ± 1,939.5 mIU/l, respectively; P < 0.01) and infant weight (6.646 ± 1.8 kg vs 5.304 ± 1.2 kg, respectively; P<0.01) in the intervention group compared to placebo. |
| Khan et al. 2018 | 5 RCTs | Term and preterm infants | Fenugreek (Trigonella foenum-graecum) | Primary outcome:  Breastmilk volume  Secondary outcome:  Maternal and neonatal safety | Fenugreek significantly increased breast milk volumes (volume per feed) compared to placebo [weighted mean difference 11.11, CI 95% 6.77 - 15.46]  No relevant data for maternal and neonatal safety was reported |
| Grzeskowiak et al. 2018 | 5 RCTs | Preterm infants | Domperidone | Primary outcome:  breastmilk volume  Secondary outcomes:  longer-term breastfeeding outcomes after completion of the RCT  Maternal and neonatal adverse events | Meta-analysis identified a moderate increase in daily breastmilk volume in those given domperidone compared to placebo of 88.3 ml/day (95% CI 56.8–119.8)  Three studies reported no significant maternal  adverse events at all in either treatment group.  Adverse events were reported in two studies, with the pooled estimate identifying no difference in prevalence between women receiving domperidone compared with placebo (RR 1.05, 95% CI 0.65–1.71). Adverse events reported included headache, gastrointestinal symptoms, respiratory symptoms and neuro-behavioural symptoms (e.g. sleep disturbance, dizziness, drowsiness or restlessness). No serious adverse effects were reported in any study. Potential cardiac adverse events were only specifically evaluated in one study. No women were identified as having a prolonged QTc interval. |
| Foong et al. 2020 | 41 RCTs | Term infants | Pharmacological galactagogues vs placebo – 9 RCTs:   - domperidone - metoclopramide - sulpiride - thyrotropin-releasing hormone)   Natural galactagogues vs placebo – 27 RCTs:   - banana flower (*Musa x paradisiaca*) - fennel (*Foeniculum vulgare*) - fenugreek (*Trigonella foenum-graecum*), - ginger (*Zingiber officinale*) - turmeric (*Curcuma longa*) - ixbut (*Euphorbia lancifolia*), - levant cotton (*Gossypium herbaceum*), - malunggay (M*oringa oleifera)* - palm dates (*Phoenix dactylifera L*.) - pork knuckle - shatavari (*Asparagus racemosus*) - milk thistle (*Silybum marianum*), - torbangun leaves (*Coleus amboinicus L.))*   One oral galactagogues vs another oral galactagogue – 8 RCTs   - Chanbao; Bue Xue Sheng Ru, - Domperidone - malunggay (*Moringa oleifera*), - fenugreek (*Trigonella foenum-graecum*) - palm dates (*Phoenix dactylifera L*.) - torbangun (*Coleus amboinicus L*.) moloco, - Mu Er Wu You, Kun Yuan Tong Ru | Primary outcomes:  Proportion of mothers who continued breastfeeding at 3, 4 and 6 months  Infant weight where the infants received only breastmilk    Volume of breastmilk at the latest time measured | Due to extremely limited, very low certainty evidence, the authors could not conclude whether galactagogues have any effect on the proportion of mothers who continued breastfeeding at 3, 4 and 6 months.  There is low-certainty evidence that pharmacological galactagogues may increase milk volume. Subgroup analyses provided some evidence that natural galactagogues may increase infant weight and milk volume, but due to heterogeneity of the studies, imprecision of measurements and incomplete reporting, they could not ascertain the magnitude of the effect.  It could noy be concluded if one galactagogue performs better than another. |
| Shen et al. 2021 | 16 RCTs | Term and preterm infants | Domperidone – 10 RCTs  Metoclopramide – 6 RCTs | Primary outcomes:  change in daily breastmilk volume  maternal and neonatal side effects  Secondary outcomes:  Differences in daily breastmilk volume between the domperidone and metoclopramide groups  Differences in maternal and neonatal side effects between the domperidone and metoclopramide groups | Domperidone:   - In mothers of preterm infants, domperidone demonstrated a significant increase in daily milk volume (MD = 90.53 mL/day, 95% CI [65.42 to 115.64]). Metoclopramide did not show significant difference in daily milk volume in women with preterm infants (MD = -1.14 mL/day, 95% CI [-31.42 to 29.14]). - Nine studies of domperidone assessed maternal or neonatal side effects or both. Five of these studies showed no adverse side effects of the domperidone intervention. The remaining four studies reported adverse maternal side effects during domperidone intervention (GI disturbance, headaches, dizziness, dry mouth). Only one of these studies assessed QT prolongation as a side effect. There were no reported cases of prolonged QTc syndrome in mothers. Five infants were found to have prolonged QTc syndrome, but all were asymptomatic and did not require treatment.   Metoclopramide:   - Six studies looking at metoclopramide assessed maternal side effects or neonatal side effects or both. - Of the six studies, only one study reported no maternal and neonatal side effects during the metoclopramide intervention. - Adverse maternal side effects were noted in other five studies. Central nervous system symptoms (e.g., headache) and psychological symptoms (e.g., anxiety, depression) were commonly reported maternal side effects.   No differences in maternal side effects were noted with domperidone (RR= 1.20, 95% CI [0.74 to 1.97], or metoclopramide (RR= 1.05, 95% CI [0.52 to 2.11], in women with preterm infants.  There was insufficient data for women with term infants in this review for conclusions to be drawn. |
| Kwan & Abdul‑Rahman 2021 | 13 RCTs | Term and preterm infants | Fenugreek (*Trigonella foenum-graecum)* – 4 RCTs  Goat’s rue (*Galega officinalis*) and milk thistle (silymarin-phosphatidylserine) – 2 RCTs  Milk thistle (*Silybum marianum)* alone – 1 RCT  Milk thistle (*Silybum marianum)* and Carduus (*Cardus marianus L.) –* 1 RCT  Herbal tea mixture containing stinging nettle (*Urtica diocia L*.), Melissa (*Melisa officinalis L*.), caraway (*Carum carvi L*.), anise (*Pimpinella anisum*), fennel (*Foeniculi vulgare Mill*), Goat’s Rue (*Galega offcinalis*) and lemon grass (*Cymbopogon citratus*) – 1 RCT  Banana flower of *Musa x paradisiaca sp.* – 1 RCT  Ginger (*Zingiber officinale*) – 1 RCT  Malunggay (*Moringa oleifera*) – 1 RCT  Shatavari (*Asparagus racemosus*) – 1 RCT | Breastmilk volume  Infant weight  Prolactin levels | Fenugreek:   - Two RCTs showed a statistically significant increase in breastmilk production and infant weight - One RCT found a statistically significant increase in infant weight. - Only one of the four studies assessed prolactin levels, there was no significant difference between placebo and intervention group.   Goat’s rue and milk thistle:   - Both RCTs showed a statistically significant increase in milk volumes in the intervention group compared to placebo.   Milk thistle alone: statistically significant increase in breastmilk volumes in the intervention group compared to placebo.  Milk thistle and carduus: no statistically significant increase in breastmilk volumes compared to placebo.  Herbal tea mixture: statistically significant increase in breastmilk volumes in the intervention group compared to placebo.  Banana flower: statistically significant increase in breastmilk volumes in the intervention group compared to placebo.  Ginger: statistically significant increase in breastmilk volumes in the intervention group compared to placebo at the 3^rd^ day of intervention, but by the 7^th^ day volumes were similar. Mean serum prolactin levels were similar between groups.  Malunggay: there was an increasing trend in milk production in those given the intervention compared to placebo.  Shatavari: there was a threefold increase in prolactin levels in those given the intervention compared to placebo. |
| Dilokthornsakul et al. 2022 | 5 RCTs | Term infants | Ginger alone (*Zingiber officinale*) – 2 RCTs  Ginger in combination with other herbs – 3 RCTs:   - Mixed herbs Xiong-gui-tiao-xue-yin which included ginger (Japanese angelica root, Cnidium rhizome, Rehmannia root, Atractylodes rhizome, hoelen, Citrus unshiu peel, Cyperus rhizome, Moutan bark, Lindera root, jujube fruit, Siberian motherwort herb, ginger rhizome, and Glycyrrhiza root) – 1 RCT - Ginger with pandan (*Pandanus amaryllifolius*) – 1 RCT - Ginger with turmeric (*Curcuma longa*) and fenugreek (*Trigonella foenum-graecum*) – 1 RCT | Primary outcome:  Breastmilk volume  Secondary outcomes:  serum prolactin and oxytocin  milk nutrient contents | Ginger alone:   - One RCT indicated that ginger at a dose of 1,000 mg/day significantly increased 24-h human milk volume compared to placebo on day 3 after delivery, regardless of delivery method (191.0 ± 71.2 vs. 135.0 ± 61.5 mL, p < 0.001). However, no difference was observed on day 7. - The second RCT on ginger alone showed that ginger instant powder at a dose of 10 g/day did not significantly increase 24-h human milk volume compared to placebo on days 2–3 after delivery (95.0 ± 104.3 vs. 88.7 ± 91.7 mL, p = 0.679) in mothers who had undergone a C-section.   Ginger in combination products:  Ginger in combination products increased milk volume   - mixed herbs (Xiong-gui-tiao-xue-yin) enhanced 24-h human milk volume on day 6 postpartum (413.7 ± 68.1 vs. 293.3 ± 98.5 g, p = 0.046) - The study of ginger in combination with pandan evaluated how many drops of breastmilk that could be squeezed from breasts by nurse practitioners 72h postpartum, nurses then recorded a score. Mothers in the ginger plus pandan solution group had higher human milk volume scores than mothers in the pandan alone group (1.9 vs. 1.6, p = not reported) - Ginger in combination with fenugreek and turmeric increased 24-h human milk volume at week 2 (1030.0 ± 264.0 vs. 805.0 ± 181.0 mL, p = 0.003) and week 4 (1399.0 ± 312.0 vs. 896.0 ± 185.0 mL, p < 0.001) postpartum compared to placebo.   Prolactin levels were not different between women given ginger and those given placebo on day 3 after delivery (p = 0.740) |
