# Supplementary material for: Herbal galactagogues to improve breastmilk production and lactation in mothers of preterm babies: a systematic review of clinical trials
Source: Eur J Clin Nutr. 2025 Dec 5;80(2):146–58. doi: 10.1038/s41430-025-01679-x (PMC12929060; doi:10.1038/s41430-025-01679-x)
Supplement: Supplementary file 2 — Supplementary Table 2 [file 41430_2025_1679_MOESM2_ESM.docx]

# Supplementary Table 2: Complete Search Strategy

## MEDLINE

Ovid MEDLINE(R) ALL <1946 to August 15, 2024>

1. exp Infant, Premature/ or exp Premature Birth/ or prematur$.mp. 259951
2. preterm infant.mp. 6429
3. NICU.mp. or Intensive Care, Neonatal/ or Intensive Care Units, Neonatal/ 31776
4. (neonatal adj3 unit*).mp. [mp=title, book title, abstract, original title, name of substance word, subject heading word, floating sub-heading word, keyword heading word, organism

supplementary concept word, protocol supplementary concept word, rare disease supplementary concept word, unique identifier, synonyms, population supplementary concept word, anatomy supplementary concept word] 37347

1. low birth weight.mp. or exp Infant, Low Birth Weight/ 56669
2. VLBW.mp. or Infant, Very Low Birth Weight/ 11680
3. Infant, Extremely Low Birth Weight/ or ELBW.mp. 3144
4. (nursing adj2 (maternal$ or mother$ or baby or babies or infant$ or newborn$ or neonat$ or neo nat$ or perinat$ or peri nat$ or premie or premies)).ti,ab. 4323

9 1 or 2 or 3 or 4 or 5 or 6 or 7 or 8 318520

1. breast feeding.mp. or exp Breast Feeding/ 51448
2. breastfe$.ti,ab. 40901
3. (breast$ adj2 (fed$ or feed$)).ti,ab. 19971
4. lactation.mp. or exp Lactation/ 73123
5. Milk, Human/ or Breast Milk Expression/ or Milk Ejection/ 24312
6. (breastmilk$ or lactat$).ti,ab. 197193
7. ((breast$ or mother$ or human or maternal$) adj2 milk).ti,ab. 31138
8. maternal breastmilk.ti,ab. 29
9. ((milk$ or breast$) adj2 express$).ti,ab. 10834
10. (breast pump$ or breastpump$).ti,ab. 479
11. (hand$ adj2 express$).ti,ab. 2427
12. ((express$ or extract$) adj2 milk).ti,ab. 2620 22 pump.ti,ab. 118492

23 (milk$ adj2 (suppl$ or volume$ or quantity or yield$)).ti,ab. 15300

24 10 or 11 or 12 or 13 or 14 or 15 or 16 or 17 or 18 or 19 or 20 or 21 or 22 or 23 421851

25 9 and 24 14998

1. "randomized controlled trial".pt. 619082
2. (random$ or placebo$ or single blind$ or double blind$ or triple blind$).ti,ab. 1651179
3. ((singl$ or doubl$ or trebl$ or tripl$) adj (blind$3 or mask$3)).tw. 207254
4. randomi?ed control$ trial$.tw. 288317
5. placebo$.ti,ab. 258935

31 random$.ti,ab. 1543377

32 clinical trial.mp. or exp Clinical Trial/ 1132915

33 26 or 27 or 28 or 29 or 30 or 31 or 32 2169993

34 33 and 25 2173

1. galactogogue$.mp. or exp Galactogogues/ 243
2. galactagogue.mp. 136
3. Plants, Medicinal/ or lactogogue.mp. 63447
4. exp Plant Extracts/ or lactagogue.mp. 218371
5. lactogenic.mp. 1640
6. galactagogic.mp. 3
7. promot$ lactat$.ti,ab. 168
8. stimula$ lactat$.ti,ab. 204
9. enhanc$ lactat$.ti,ab. 297
10. (insufficie$ adj2 milk$).mp. [mp=title, book title, abstract, original title, name of substance word, subject heading word, floating sub-heading word, keyword heading word, organism

supplementary concept word, protocol supplementary concept word, rare disease supplementary concept word, unique identifier, synonyms, population supplementary concept word, anatomy supplementary concept word] 593

1. (herb$ adj2 galact$).mp. [mp=title, book title, abstract, original title, name of substance word, subject heading word, floating sub-heading word, keyword heading word, organism

supplementary concept word, protocol supplementary concept word, rare disease supplementary concept word, unique identifier, synonyms, population supplementary concept word, anatomy supplementary concept word] 46

1. (Fenugreek or Trigonella foenumgraecum).mp. 1442
2. (Shatavari or Asparagus racemosus).mp. 235
3. (Torbangun or Coleus amboinicus lour).mp. 19
4. (fennel or foeniculum vulgare).mp. 1231
5. (milk thistle or silybum marianum or silymarin phosphatidylserine).mp. 1690
6. (Chasteberry or vitex agnus castus).mp. 257
7. (Goat's rue or Galega officinalis).mp. 99
8. (Mulunggay or moringa oleifera).mp. 2096
9. (Carduus or Cardus marianus).mp. 133
10. (stinging nettle or urtica diocia).mp. 286
11. (Melissa or melisa officinalis).mp. 888
12. (caraway or carum carvi).mp. 620
13. (anise or pimpinella anisum).mp. 762
14. (fennel or foeniculi vulgare mill).mp. 939
15. (lemon grass or cymbopogon citratus).mp. 679
16. (banana flower or musa x paradisiaca).mp. 49
17. (ginger or zingiber officinale).mp. 5616
18. (blackseed or Nigella sativa).mp. 2219
19. (coriander or cilantro or Coriandrum sativum).mp. 1242
20. (dill or Anethum graveolens).mp. 663
21. (Alfalfa or Medicago sativa).mp. 11020
22. (Blessed thistle or Cnicus benedictus).mp. 34
23. (red clover or Trifolium pratense).mp. 1644
24. (Chandrashoor or Lepidium sativum).mp. 850
25. (Herb$ or spic$).mp. [mp=title, book title, abstract, original title, name of substance word, subject heading word, floating sub-heading word, keyword heading word, organism supplementary concept word, protocol supplementary concept word, rare disease supplementary concept word, unique identifier, synonyms, population supplementary concept word, anatomy supplementary concept word] 215942
26. Phytotherapy.mp. or exp Phytotherapy/ 43914
27. Ayurvedic medicine.mp. or exp Medicine, Ayurvedic/ 3066
28. Traditional Chinese Medicine.mp. or exp Medicine, Chinese Traditional/ 50714
29. traditional medicine.mp. or Medicine, Traditional/ 27149
30. diet therapy.mp. or Diet Therapy/ 67680
31. medicinal teas.mp. or Teas, Medicinal/ 63
32. ((herbal or Chinese or ayurved$) adj2 (therap$ or medicine? or treatment? or intervention?)).ti,ab. 72533
33. (Raspberry leaf or Rubus idaeus).mp. 387
34. (Damiana or Turnera diffusa).mp. 105
35. 35 or 36 or 37 or 38 or 39 or 40 or 41 or 42 or 43 or 44 or 45 or 46 or 47 or 48 or 49 or 50 or 51 or 52 or 53 or 54 or 55 or 56 or 57 or 58 or 59 or 60 or 61 or 62 or 63 or 64 or 65 or 66 or 67 or 68 or 69 or 70 or 71 or 72 or 73 or 74 or 75 or 76 or 77 or 78 or 79 547332

81 34 and 80 95

## Embase Classic + Embase

<1947 to 2024 August 16>

1. exp prematurity/ or exp premature labor/ or prematur*.mp. 400896
2. exp newborn intensive care/ or exp neonatal intensive care unit/ or NICU.mp. 64234
3. (neonatal adj3 unit*).mp. [mp=title, abstract, heading word, drug trade name, original title, device manufacturer, drug manufacturer, device trade name, keyword heading word, floating subheading word, candidate term word]52644
4. low birth weight.mp. or exp low birth weight/ 76140
5. VLBW.mp. or exp very low birth weight/ 19508
6. ELBW.mp. or exp extremely low birth weight/ 5146
7. (nursing adj2 (maternal$ or mother$ or baby or babies or infant$ or newborn$ or neonat$ or neo nat$ or perinat$ or perinat$ or premie or premies)).mp. 8176

8 1 or 2 or 3 or 4 or 5 or 6 or 7 484486

1. breast feeding.mp. or exp breast feeding/ 78749
2. breastfe$.mp. 53423
3. (breast$ adj2 (fed$ or feed$)).mp. 83005
4. exp lactation/ or lactation.mp. 85608
5. human milk.mp. or breast milk/ 43236
6. breast milk expression.mp. or exp breast milk expression/ 636
7. (breastmilk$ or lactat$).mp. 393105
8. ((breast$ or mother$ or human or maternal$) adj2 milk).mp. 51306
9. maternal breastmilk.mp. 44
10. ((milk$ or breast$) adj2 express$).mp. 15683
11. (breast pump$ or breastpump$).mp. 977
12. (hand$ adj2 express$).mp. 3461
13. (express$ or extract$).mp. 6541168

22 pump.mp. 237748

23 (milk$ adj2 (suppl$ or volume$ or quantity or yield$)).ti,ab. 16963

24 9 or 10 or 11 or 12 or 13 or 14 or 15 or 16 or 17 or 18 or 19 or 20 or 21 or 22 or 23

7151839

1. random$ controlled trial.mp. or exp "randomized controlled trial (topic)"/ 1146813
2. (random$ or placebo$ or single blind$ or double blind$ or triple blind$).ti,ab. 2283327
3. ((singl$ or doubl$ or trebl$ or tripl$) adj (blind$3 or mask$3)).tw. 300209
4. randomi?ed control$ trial$.tw. 376341
5. placebo$.ti,ab. 390026

30 random$.ti,ab. 2124188

31 clinical trial.mp. or exp clinical trial/ 2384716

32 25 or 26 or 27 or 28 or 29 or 30 or 31 3761419

1. galactogogue.mp. or exp galactogogue/ 342
2. galactagogue.mp. 190
3. exp Asparagus racemosus extract/ or exp herbaceous agent/ or lactogogue.mp. or exp herb/ 74306
4. exp Nigella sativa extract/ or lactagogue.mp. 1443
5. exp plant extract/ or lactogenic.mp. 293894
6. medicinal plant.mp. or exp medicinal plant/ 337194
7. galactagogic.mp. 5
8. promot$ lactat$.mp. 203
9. stimula$ lactat$.mp. 268
10. enhanc$ lactat$.mp. 365
11. (insufficie$ adj2 milk*).mp. [mp=title, abstract, heading word, drug trade name, original title, device manufacturer, drug manufacturer, device trade name, keyword heading word, floating

subheading word, candidate term word]723

1. (herb* adj2 galact*).mp. [mp=title, abstract, heading word, drug trade name, original title, device manufacturer, drug manufacturer, device trade name, keyword heading word, floating subheading word, candidate term word]69
2. (Fenugreek or Trigonella foenumgraecum).mp. 3665
3. (Shatavari or Asparagus racemosus).mp. 942
4. (Torbangun or Coleus amboinicus lour).mp. 40
5. (fennel or foeniculum vulgare).mp. 3013
6. (milk thistle or silybum marianum or silymarin phosphatidylserine).mp. 3344
7. (Chasteberry or vitex agnus castus).mp. 770
8. (Goat's rue or Galega officinalis).mp. 160
9. (Mulunggay or moringa oleifera).mp. 4106
10. (Carduus or Cardus marianus).mp. 226
11. (stinging nettle or urtica diocia).mp. 378
12. (Melissa or melisa officinalis).mp. 2482
13. (caraway or carum carvi).mp. 1090
14. (anise or pimpinella anisum).mp. 1662
15. (fennel or foeniculi vulgare mill).mp. 2702
16. (lemon grass or cymbopogon citratus).mp. 1880
17. (banana flower or musa x paradisiaca).mp. 431
18. (ginger or zingiber officinale).mp. 11932
19. (blackseed or Nigella sativa).mp. 3774
20. (coriander or cilantro or Coriandrum sativum).mp. 2439
21. (dill or Anethum graveolens).mp. 1457
22. (Alfalfa or Medicago sativa).mp. 11587
23. (Blessed thistle or Cnicus benedictus).mp. 67
24. (red clover or Trifolium pratense).mp. 2235
25. (Chandrashoor or Lepidium sativum).mp. 1235
26. (Herb* or spic*).mp. [mp=title, abstract, heading word, drug trade name, original title, device manufacturer, drug manufacturer, device trade name, keyword heading word, floating subheading word, candidate term word]276057
27. Phytotherapy.mp. or exp phytotherapy/ 21351
28. Ayurved$.mp. or exp Ayurveda/ 15981
29. Traditional Chinese Medicine.mp. or exp Chinese medicine/ 98778
30. traditional medicine.mp. or exp traditional medicine/ 164954
31. diet therapy.mp. or exp diet therapy/ 462109
32. (medicinal adj2 (tea or teas)).mp. or exp medicinal tea/ 239
33. ((herbal or Chinese or ayurved*) adj2 (therap* or medicine? or treatment? or intervention?)).mp. 142042
34. (Raspberry leaf or Rubus idaeus).mp. 450
35. (Damiana or Turnera diffusa).mp. 178
36. 33 or 34 or 35 or 36 or 37 or 38 or 39 or 40 or 41 or 42 or 43 or 44 or 45 or 46 or 47 or 48 or 49 or 50 or 51 or 52 or 53 or 54 or 55 or 56 or 57 or 58 or 59 or 60 or 61 or 62 or 63 or 64 or 65 or 66 or 67 or 68 or 69 or 70 or 71 or 72 or 73 or 74 or 75 or 76 or 77 or 78 1245307

80 8 and 24 and 32 and 79 1254

## Cochrane

Date Run: 19/08/2024 14:33:49

ID Search Hits

#1 MeSH descriptor: [Premature Birth] this term only 2519

#2 MeSH descriptor: [Infant, Very Low Birth Weight] explode all trees 1414

#3 MeSH descriptor: [Infant, Low Birth Weight] explode all trees 3058

#4 MeSH descriptor: [Intensive Care, Neonatal] 1 tree(s) exploded 428

#5 MeSH descriptor: [Intensive Care Units, Neonatal] explode all trees 1290

#6 (neonatal NEAR/3 unit*):ti,ab,kw 5785

#7 MeSH descriptor: [Infant, Extremely Low Birth Weight] explode all trees 183 #8 "ELBW":ti,ab,kw 275

#9 "VLBW":ti,ab,kw 1053

#10 (nursing NEAR/2 (maternal* or mother* or baby or babies or infant* or newborn* or

neonat* or (neo NEXT nat*) or perinat* or (peri NEXT nat*) or premie or premies)):ti,ab,kw 723

#11 {OR #1-#10} 11665

| #12 | MeSH descriptor: [Breast Feeding] explode all trees | 2935 |
| --- | --- | --- |
| #13 | MeSH descriptor: [Breast Feeding] explode all trees | 2935 |
| #14 | breastfee*:ti,ab,kw 8374 |  |
| #15 | (breast* NEAR/2 (fed* or feed*)):ti,ab,kw 7645 |  |
| #16 | MeSH descriptor: [Lactates] explode all trees 4028 |  |
| #17 | MeSH descriptor: [Milk, Human] explode all trees | 1526 |

#18 MeSH descriptor: [Breast Milk Expression] explode all trees 44

#19 MeSH descriptor: [Milk Ejection] explode all trees 17

| #20 | (breastmilk* or lactat*):ti,ab,kw 20441 |  |
| --- | --- | --- |
| #21 | ((breast* or mother* or human or maternal*) NEAR/2 milk):ti,ab,kw | 5233 |
| #22 | (mothers NEXT own NEXT milk):ti,ab,kw 36 |  |
| #23 | (mothers NEXT milk):ti,ab,kw 80 |  |
| #24 | (maternal NEXT breast NEXT milk):ti,ab,kw 62 |  |
| #25 | (maternal NEXT breastmilk):ti,ab,kw 6 |  |

#26 (mother* NEAR/2 milk):ti,ab,kw 701

#27 ((milk* or breast*) NEAR/2 express*) 820

#28 (breast NEXT pump* or breastpump*):ti,ab,kw 169 #29 (hand* NEAR/2 express*):ti,ab,kw 120

#30 (express* or extract*):ti,ab,kw 125445

#31 pump:ti,ab,kw 16389

#32 (milk* NEAR/2 (suppl* or volume* or quantity or yield*)):ti,ab,kw 1186

#33 {OR #12-#32} 171145

#34 (randomi?ed NEXT control* NEXT trial*):ti,ab,kw 714856

#35 MeSH descriptor: [Randomized Controlled Trial] explode all trees 37

#36 (random* or placebo* or (single NEXT blind*) or (double NEXT blind*) or (triple NEXT blind*)):ti,ab,kw 1501658

#37 ((singl* or doubl* or trebl* or tripl*) NEAR/1 (blind* or mask*)):ti,ab,kw 429058

| #38 | (placebo*):ti,ab,kw | 394303 |
| --- | --- | --- |
| #39 | (random*):ti,ab,kw | 1360830 |

#40 MeSH descriptor: [Clinical Trial] explode all trees45 #41 {OR #34-#40} 1501874

| #42 | MeSH descriptor: [Galactogogues] explode all trees | 20 |
| --- | --- | --- |
| #43 | Galactagogues:ti,ab,kw 12 |  |
| #44 | MeSH descriptor: [Plants, Medicinal] explode all trees | 1149 |
| #45 | lactogogue:ti,ab,kw 3 |  |
| #46 | lactogenic:ti,ab,kw 10 |  |
| #47 | (promot* NEXT lactat*):ti,ab,kw 19 |  |
| #48 | (stimula* NEXT lactat*):ti,ab,kw 6 |  |
| #49 | (enhanc* NEXT lactat*):ti,ab,kw 14 |  |
| #50 | (insufficie* NEAR/2 milk*):ti,ab,kw 125 |  |
| #51 | (herb* NEAR/2 galact*):ti,ab,kw 19 |  |
| #52 | ("Raspberry leaf" or "Rubus idaeus"):ti,ab,kw 20 |  |
| #53 | (Damiana or "Turnera diffusa"):ti,ab,kw 23 |  |
| #54 | (Shatavari or "Asparagus racemosus"):ti,ab,kw 121 |  |
| #55 | (Torbangun or "Coleus amboinicus lour"):ti,ab,kw | 7 |

#56 (fennel or "foeniculi vulgare mill"):ti,ab,kw 222

#57 ("milk thistle" or "silybum marianum" or "silymarin phosphatidylserine"):ti,ab,kw 229

#58 (Chasteberry or "vitex agnus castus"):ti,ab,kw 87

#59 ("Goat's rue" or "Galega officinalis"):ti,ab,kw 2

#60 (Mulunggay or "moringa oleifera"):ti,ab,kw 170

#61 (Carduus or "Cardus marianus"):ti,ab,kw3

#62 ("stinging nettle" or "urtica diocia"):ti,ab,kw 29

#63 (Melissa or "melisa officinalis"):ti,ab,kw 223

#64 (caraway or "carum carvi"):ti,ab,kw 84

#65 (anise or "pimpinella anisum"):ti,ab,kw 93

#66 ("lemon grass" or "cymbopogon citratus"):ti,ab,kw 38

#67 ("banana flower" or "musa x paradisiaca"):ti,ab,kw 9

#68 (ginger or "zingiber officinale"):ti,ab,kw 1338

#69 (blackseed or "Nigella sativa"):ti,ab,kw 407

#70 (coriander or cilantro or "Coriandrum sativum"):ti,ab,kw 98 #71 (dill or "Anethum graveolens"):ti,ab,kw 105

#72 (Alfalfa or "Medicago sativa"):ti,ab,kw 134

#73 ("Blessed thistle" or "Cnicus benedictus"):ti,ab,kw 1

#74 ("red clover" or "Trifolium pratense"):ti,ab,kw 118

#75 (Chandrashoor or "Lepidium sativum"):ti,ab,kw 11

#76 (Herb* or spic*):ti,ab,kw 15927

#77 MeSH descriptor: [Phytotherapy] explode all trees 5067

#78 MeSH descriptor: [Medicine, Ayurvedic] explode all trees 130

#79 MeSH descriptor: [Medicine, Chinese Traditional] explode all trees 1781

#80 MeSH descriptor: [Medicine, Traditional] explode all trees 2268

#81 MeSH descriptor: [Diet Therapy] explode all trees 8315

#82 MeSH descriptor: [Teas, Medicinal] this term only 1

#83 ((herbal or Chinese or ayurved*) NEAR/2 (therap* or medicine* or treatment* or intervention*)):ti,ab,kw 20491

#84 {OR #42-#83} 42830

#85 #11 AND #33 AND #41 AND #84 42

|  | CINAHL |  |  |  |
| --- | --- | --- | --- | --- |
| **#** | **Query** | **Limiters/Expanders** | **Last Run Via** | **Results** |
| S74 | S6 AND S71 AND S72 AND S73 | Expanders - Apply equivalent subjects  Search modes - Find all my search terms | Interface - EBSCOhost Research Databases  Search Screen - Advanced Search Database - CINAHL Plus with Full Text | 63 |
| S73 | S27 OR S28 OR S29 OR S30 OR S31 OR S32 OR S33 OR S34 OR S35 OR S36 OR S37 OR S38 OR S39 OR S40 OR S41 OR S42 OR S43 OR S44 OR S45 OR S46 OR S47 OR S48 OR S49 OR S50 OR S51 OR S52 OR S53 OR S54 OR S55 OR S56 OR S57 OR S58 OR S59 OR S60 OR S61 OR S62 OR S63 OR S64 OR S65 OR S66 OR S67 OR S68 OR S69 OR S70 | Expanders - Apply equivalent subjects  Search modes - Find all my search terms | Interface - EBSCOhost Research Databases  Search Screen - Advanced Search Database - CINAHL Plus with Full Text | 178,110 |
| S72 | S20 OR S21 OR S22 OR S23 OR S24 OR S25 OR S26 | Expanders - Apply equivalent subjects  Search modes - Find all my search terms | Interface - EBSCOhost Research Databases  Search Screen - Advanced Search Database - CINAHL Plus with Full Text | 698,644 |
| S71 | S7 OR S8 OR S9 OR S10 OR S11 OR S12 OR S13 OR S14 OR S15 OR S16 OR S17 OR S18 OR S19 | Expanders - Apply equivalent subjects  Search modes - Find all my search terms | Interface - EBSCOhost Research Databases  Search Screen - Advanced Search Database - CINAHL Plus with Full Text | 483,715 |
| S70 | ((herbal or Chinese or ayurved*) N2 (therap* or medicine* or treatment* or intervention*)) | Expanders - Apply equivalent subjects  Search modes - Find all my search terms | Interface - EBSCOhost Research Databases  Search Screen - Advanced Search Database - CINAHL Plus with Full Text | 42,789 |
| S69 | "medicinal teas" | Expanders - Apply equivalent subjects  Search modes - Find all my search terms | Interface - EBSCOhost Research Databases  Search Screen - Advanced Search Database - CINAHL Plus with Full Text | 20 |
| S68 | (MH "Diet Therapy") OR "diet therapy" OR "nutrition intervention" | Expanders - Apply equivalent subjects  Search modes - Find all my search terms | Interface - EBSCOhost Research Databases  Search Screen - Advanced Search Database - CINAHL Plus with Full Text | 30,029 |
| S67 | (MH "Medicine, Traditional")  OR (MH "Traditional Healers") OR "traditional medicine" | Expanders - Apply equivalent subjects  Search modes - Find all my search terms | Interface - EBSCOhost Research Databases  Search Screen - Advanced Search  Database - CINAHL Plus with Full Text | 11,484 |

| S66 | (MH "Medicine, Ayurvedic") OR ayurvedic | Expanders - Apply equivalent subjects  Search modes - Find all my search terms | Interface - EBSCOhost Research Databases  Search Screen - Advanced Search Database - CINAHL Plus with Full Text | 2,344 |
| --- | --- | --- | --- | --- |
| S65 | (MH "Medicine, Herbal") OR (MH "Medicine, East Asian Traditional") OR (MH "Medicine, Chinese Traditional") OR (MH "Medicine, Persian") OR (MH "Medicine, Latin American Traditional") OR (MH "Medicine, Native American Traditional") OR (MH "Medicine, African Traditional") OR "phytotherapy or herbal medicine" | Expanders - Apply equivalent subjects  Search modes - Find all my search terms | Interface - EBSCOhost Research Databases  Search Screen - Advanced Search Database - CINAHL Plus with Full Text | 30,390 |
| S64 | "Herb* or spic*" | Expanders - Apply equivalent subjects  Search modes - Find all my search terms | Interface - EBSCOhost Research Databases  Search Screen - Advanced Search Database - CINAHL Plus with Full Text | 125 |
| S63 | "Chandrashoor" or "Lepidium sativum" | Expanders - Apply equivalent subjects  Search modes - Find all my search terms | Interface - EBSCOhost Research Databases  Search Screen - Advanced Search Database - CINAHL Plus with Full Text | 38 |
| S62 | "red clover" or "Trifolium pratense" | Expanders - Apply equivalent subjects  Search modes - Find all my search terms | Interface - EBSCOhost Research Databases  Search Screen - Advanced Search Database - CINAHL Plus with Full Text | 239 |
| S61 | "Blessed thistle" or "Cnicus benedictus" | Expanders - Apply equivalent subjects  Search modes - Find all my search terms | Interface - EBSCOhost Research Databases  Search Screen - Advanced Search Database - CINAHL Plus with Full Text | 187 |
| S60 | "Alfalfa" or "Medicago sativa" | Expanders - Apply equivalent subjects  Search modes - Find all my search terms | Interface - EBSCOhost Research Databases  Search Screen - Advanced Search Database - CINAHL Plus with Full Text | 121 |
| S59 | "dill" or "Anethum graveolens" | Expanders - Apply equivalent subjects  Search modes - Find all my search terms | Interface - EBSCOhost Research Databases  Search Screen - Advanced Search Database - CINAHL Plus with Full Text | 119 |
| S58 | "coriander" or "cilantro" or "Coriandrum sativum" | Expanders - Apply equivalent subjects  Search modes - Find all my  search terms | Interface - EBSCOhost Research Databases  Search Screen - Advanced Search | 195 |

|  |  |  | Database - CINAHL Plus with Full  Text |  |
| --- | --- | --- | --- | --- |
| S57 | "blackseed" or "Nigella sativa" | Expanders - Apply equivalent subjects  Search modes - Find all my search terms | Interface - EBSCOhost Research Databases  Search Screen - Advanced Search Database - CINAHL Plus with Full Text | 418 |
| S56 | "ginger" or "zingiber officinale" | Expanders - Apply equivalent subjects  Search modes - Find all my search terms | Interface - EBSCOhost Research Databases  Search Screen - Advanced Search Database - CINAHL Plus with Full Text | 2,029 |

| S55 | "banana flower" or "musa x paradisiaca" | Expanders - Apply equivalent subjects  Search modes - Find all my search terms | Interface - EBSCOhost Research Databases  Search Screen - Advanced Search Database - CINAHL Plus with Full Text | 6 |
| --- | --- | --- | --- | --- |
| S54 | "lemon grass" or "cymbopogon citratus" | Expanders - Apply equivalent subjects  Search modes - Find all my search terms | Interface - EBSCOhost Research Databases  Search Screen - Advanced Search Database - CINAHL Plus with Full Text | 182 |
| S53 | "anise" or "pimpinella anisum" | Expanders - Apply equivalent subjects  Search modes - Find all my search terms | Interface - EBSCOhost Research Databases  Search Screen - Advanced Search Database - CINAHL Plus with Full Text | 132 |
| S52 | "caraway" or "carum carvi" | Expanders - Apply equivalent subjects  Search modes - Find all my search terms | Interface - EBSCOhost Research Databases  Search Screen - Advanced Search Database - CINAHL Plus with Full Text | 82 |
| S51 | "Melissa" or "melisa officinalis" | Expanders - Apply equivalent subjects  Search modes - Find all my search terms | Interface - EBSCOhost Research Databases  Search Screen - Advanced Search Database - CINAHL Plus with Full Text | 401 |
| S50 | "stinging nettle" or "urtica diocia" | Expanders - Apply equivalent subjects  Search modes - Find all my search terms | Interface - EBSCOhost Research Databases  Search Screen - Advanced Search Database - CINAHL Plus with Full Text | 188 |

| S49 | "Carduus" or "Cardus marianus" | Expanders - Apply equivalent subjects  Search modes - Find all my search terms | Interface - EBSCOhost Research  Databases  Search Screen - Advanced Search Database - CINAHL Plus with Full Text | 8 |
| --- | --- | --- | --- | --- |
| S48 | "Mulunggay" or "moringa oleifera" | Expanders - Apply equivalent subjects  Search modes - Find all my search terms | Interface - EBSCOhost Research Databases  Search Screen - Advanced Search Database - CINAHL Plus with Full Text | 319 |
| S47 | "Goat's rue" or "Galega officinalis" | Expanders - Apply equivalent subjects  Search modes - Find all my search terms | Interface - EBSCOhost Research Databases  Search Screen - Advanced Search Database - CINAHL Plus with Full Text | 11 |
| S46 | "Chasteberry" or "vitex agnus castus" | Expanders - Apply equivalent subjects  Search modes - Find all my search terms | Interface - EBSCOhost Research Databases  Search Screen - Advanced Search Database - CINAHL Plus with Full Text | 97 |
| S45 | "milk thistle" or "silybum marianum" or "silymarin phosphatidylserine" | Expanders - Apply equivalent subjects  Search modes - Find all my search terms | Interface - EBSCOhost Research Databases  Search Screen - Advanced Search Database - CINAHL Plus with Full Text | 539 |
| S44 | ("fennel" or "foeniculum vulgare mill") | Expanders - Apply equivalent subjects  Search modes - Find all my search terms | Interface - EBSCOhost Research Databases  Search Screen - Advanced Search Database - CINAHL Plus with Full Text | 296 |
| S43 | "fennel" or "foeniculum vulgare" | Expanders - Apply equivalent subjects  Search modes - Find all my search terms | Interface - EBSCOhost Research Databases  Search Screen - Advanced Search Database - CINAHL Plus with Full Text | 334 |
| S42 | "Torbangun" or "Coleus amboinicus lour" | Expanders - Apply equivalent subjects  Search modes - Find all my search terms | Interface - EBSCOhost Research Databases  Search Screen - Advanced Search Database - CINAHL Plus with Full Text | 4 |
| S41 | "Shatavari" or "Asparagus racemosus" | Expanders - Apply equivalent subjects  Search modes - Find all my search terms | Interface - EBSCOhost Research Databases  Search Screen - Advanced Search Database - CINAHL Plus with Full Text | 38 |
| S40 | "Fenugreek" OR "Trigonella foenumgraecum" | Expanders - Apply equivalent subjects  Search modes - SmartText Searching | Interface - EBSCOhost Research Databases  Search Screen - Advanced Search Database - CINAHL Plus with Full Text | 2 |

| S39 | "Damiana" OR "Turnera diffusa" | Expanders - Apply equivalent subjects  Search modes - SmartText Searching | Interface - EBSCOhost Research  Databases  Search Screen - Advanced Search Database - CINAHL Plus with Full Text | 27 |
| --- | --- | --- | --- | --- |
| S38 | "Raspberry leaf" OR "Rubus idaeus" | Expanders - Apply equivalent subjects  Search modes - SmartText Searching | Interface - EBSCOhost Research Databases  Search Screen - Advanced Search Database - CINAHL Plus with Full Text | 40 |
| S37 | (herb* N2 galact*) | Expanders - Apply equivalent subjects  Search modes - Find all my search terms | Interface - EBSCOhost Research Databases  Search Screen - Advanced Search Database - CINAHL Plus with Full Text | 26 |
| S36 | (insufficie* N2 milk*) | Expanders - Apply equivalent subjects  Search modes - Find all my  search terms | Interface - EBSCOhost Research Databases  Search Screen - Advanced Search Database - CINAHL Plus with Full  Text | 400 |

| S35 | "enhanc* lactat*" | Expanders - Apply equivalent subjects  Search modes - Find all my search terms | Interface - EBSCOhost Research Databases  Search Screen - Advanced Search Database - CINAHL Plus with Full Text | 36 |
| --- | --- | --- | --- | --- |
| S34 | ("stimula* lactat*") | Expanders - Apply equivalent subjects  Search modes - Find all my search terms | Interface - EBSCOhost Research Databases  Search Screen - Advanced Search Database - CINAHL Plus with Full Text | 10 |
| S33 | ("promot* lactat*") | Expanders - Apply equivalent subjects  Search modes - Find all my search terms | Interface - EBSCOhost Research Databases  Search Screen - Advanced Search Database - CINAHL Plus with Full Text | 42 |
| S32 | "galactagogic" | Expanders - Apply equivalent subjects  Search modes - Find all my search terms | Interface - EBSCOhost Research Databases  Search Screen - Advanced Search Database - CINAHL Plus with Full Text | 2 |
| S31 | "lactogenic" | Expanders - Apply equivalent subjects  Search modes - Find all my search terms | Interface - EBSCOhost Research Databases  Search Screen - Advanced Search Database - CINAHL Plus with Full Text | 20 |

| S30 | (MH "Plant Extracts+") OR (MH "Drugs, Chinese Herbal") OR "plant extract*" or "plant isolate*" | Expanders - Apply equivalent subjects  Search modes - Find all my search terms | Interface - EBSCOhost Research Databases  Search Screen - Advanced Search Database - CINAHL Plus with Full Text | 52,526 |
| --- | --- | --- | --- | --- |
| S29 | (lactogogue or lactagogue) | Expanders - Apply equivalent subjects  Search modes - Find all my search terms | Interface - EBSCOhost Research Databases  Search Screen - Advanced Search Database - CINAHL Plus with Full Text | 6 |
| S28 | (MH "Plants, Medicinal+") OR "medicinal plant*" | Expanders - Apply equivalent subjects  Search modes - Find all my search terms | Interface - EBSCOhost Research Databases  Search Screen - Advanced Search Database - CINAHL Plus with Full Text | 94,752 |
| S27 | (galactogogue or galactagogue) | Expanders - Apply equivalent subjects  Search modes - Find all my search terms | Interface - EBSCOhost Research Databases  Search Screen - Advanced Search Database - CINAHL Plus with Full Text | 145 |
| S26 | (MH "Clinical Trials+") OR "clinical trial" | Expanders - Apply equivalent subjects  Search modes - Find all my search terms | Interface - EBSCOhost Research Databases  Search Screen - Advanced Search Database - CINAHL Plus with Full Text | 388,340 |
| S25 | "random*" | Expanders - Apply equivalent subjects  Search modes - Find all my search terms | Interface - EBSCOhost Research Databases  Search Screen - Advanced Search Database - CINAHL Plus with Full Text | 536,691 |
| S24 | (MH "Placebos") OR "placebo*" | Expanders - Apply equivalent subjects  Search modes - Find all my search terms | Interface - EBSCOhost Research Databases  Search Screen - Advanced Search Database - CINAHL Plus with Full Text | 79,275 |
| S23 | ((singl* or doubl* or trebl* or tripl*) N3 (blind* or mask*)) | Expanders - Apply equivalent subjects  Search modes - Find all my search terms | Interface - EBSCOhost Research Databases  Search Screen - Advanced Search Database - CINAHL Plus with Full Text | 90,361 |

| S22 | (random* or placebo* or single blind* or double blind* or triple blind*) | Expanders - Apply equivalent subjects  Search modes - Find all my search terms | Interface - EBSCOhost Research  Databases  Search Screen - Advanced Search Database - CINAHL Plus with Full Text | 569,068 |
| --- | --- | --- | --- | --- |
| S21 | (MH "Randomized Controlled Trials+") OR (MH "Clinical Trials+") OR rct | Expanders - Apply equivalent subjects  Search modes - Find all my search terms | Interface - EBSCOhost Research Databases  Search Screen - Advanced Search Database - CINAHL Plus with Full Text | 377,530 |
| S20 | "randomised control* trial*" OR "randomized control* trial*" | Expanders - Apply equivalent subjects  Search modes - Find all my search terms | Interface - EBSCOhost Research Databases  Search Screen - Advanced Search Database - CINAHL Plus with Full Text | 236,325 |
| S19 | (milk* N2 (suppl* or volume* or quantity or yield*)) | Expanders - Apply equivalent subjects  Search modes - Find all my search terms | Interface - EBSCOhost Research Databases  Search Screen - Advanced Search Database - CINAHL Plus with Full Text | 1,658 |
| S18 | (MH "Breast Pumps") OR "pumping breast milk" | Expanders - Apply equivalent subjects  Search modes - Find all my search terms | Interface - EBSCOhost Research Databases  Search Screen - Advanced Search Database - CINAHL Plus with Full Text | 513 |
| S17 | (express* or extract*) | Expanders - Apply equivalent subjects  Search modes - Find all my search terms | Interface - EBSCOhost Research Databases  Search Screen - Advanced Search Database - CINAHL Plus with Full Text | 422,499 |
| S16 | (hand* N2 express*) | Expanders - Apply equivalent subjects  Search modes - Find all my search terms | Interface - EBSCOhost Research Databases  Search Screen - Advanced Search Database - CINAHL Plus with Full Text | 369 |
| S15 | (breast pump* or breastpump*) | Expanders - Apply equivalent subjects  Search modes - Find all my search terms | Interface - EBSCOhost Research Databases  Search Screen - Advanced Search Database - CINAHL Plus with Full Text | 1,115 |
| S14 | ((milk* or breast*) N2 express*) | Expanders - Apply equivalent subjects  Search modes - Find all my search terms | Interface - EBSCOhost Research Databases  Search Screen - Advanced Search Database - CINAHL Plus with Full Text | 3,232 |
| S13 | ((breast* or mother* or human or maternal*) N2 milk) | Expanders - Apply equivalent subjects  Search modes - Find all my search terms | Interface - EBSCOhost Research Databases  Search Screen - Advanced Search Database - CINAHL Plus with Full Text | 12,290 |

| S12 | (breastmilk* or lactat*) | Expanders - Apply equivalent subjects  Search modes - Find all my search terms | Interface - EBSCOhost Research  Databases  Search Screen - Advanced Search Database - CINAHL Plus with Full Text | 33,131 |
| --- | --- | --- | --- | --- |
| S11 | (MH "Milk, Human") OR (MH "Milk Expression") OR "human milk" OR "breast milk" | Expanders - Apply equivalent subjects  Search modes - Find all my search terms | Interface - EBSCOhost Research Databases  Search Screen - Advanced Search Database - CINAHL Plus with Full Text | 11,881 |
| S10 | (MH "Lactation") OR "lactation" | Expanders - Apply equivalent subjects  Search modes - Find all my search terms | Interface - EBSCOhost Research Databases  Search Screen - Advanced Search  Database - CINAHL Plus with Full Text | 10,431 |

| S9 | (breast* N2 (fed* or feed*)) | Expanders - Apply equivalent subjects  Search modes - Find all my search terms | Interface - EBSCOhost Research Databases  Search Screen - Advanced Search Database - CINAHL Plus with Full Text | 33,121 |
| --- | --- | --- | --- | --- |
| S8 | breastfee* | Expanders - Apply equivalent subjects  Search modes - Find all my search terms | Interface - EBSCOhost Research Databases  Search Screen - Advanced Search Database - CINAHL Plus with Full Text | 23,253 |
| S7 | (MH "Breast Feeding+") | Expanders - Apply equivalent subjects  Search modes - Find all my search terms | Interface - EBSCOhost Research Databases  Search Screen - Advanced Search Database - CINAHL Plus with Full Text | 28,520 |
| S6 | S1 OR S2 OR S3 OR S4 OR S5 | Expanders - Apply equivalent subjects  Search modes - Find all my search terms | Interface - EBSCOhost Research Databases  Search Screen - Advanced Search Database - CINAHL Plus with Full Text | 92,153 |
| S5 | (nursing N2 (maternal* or mother* or baby or babies or infant* or newborn* or neonat* or neo nat* or perinat* or per inat* or premie  or premies)). | Expanders - Apply equivalent subjects  Search modes - Find all my search terms | Interface - EBSCOhost Research Databases  Search Screen - Advanced Search  Database - CINAHL Plus with Full Text | 16,716 |

| S4 | "elbw" OR "extremely low birth weight" | Expanders - Apply equivalent subjects Search modes - SmartText Searching | Interface - EBSCOhost Research Databases  Search Screen - Advanced Search  Database - CINAHL Plus with Full Text | 711 |
| --- | --- | --- | --- | --- |
| S3 | (Neonatal N3 unit*) | Expanders - Apply equivalent subjects  Search modes - Find all my search terms | Interface - EBSCOhost Research Databases  Search Screen - Advanced Search  Database - CINAHL Plus with Full Text | 23,997 |
| S2 | (MH "Intensive Care Units, Neonatal") OR (MH "Neonatal Intensive Care Nursing") OR (MH "Intensive Care, Neonatal") OR "nicu" OR "neonatal intensive care unit*" OR "special care" OR "baby  unit*" OR "newborn intensive care" | Expanders - Apply equivalent subjects  Search modes - Find all my search terms | Interface - EBSCOhost Research Databases  Search Screen - Advanced Search  Database - CINAHL Plus with Full Text | 30,256 |

| S1 | (MH "Infant, Premature") OR (MH "Childbirth, Premature") OR (MH "Infant, Very Low Birth Weight") OR (MH "Infant, Low Birth Weight+") OR (MH "Labor, Premature") OR "premature infant*" OR "preterm infant*" OR "premature baby" OR "preterm baby" OR "premature birth" OR  "preterm birth" | Expanders - Apply equivalent subjects  Search modes - Find all my search terms | Interface - EBSCOhost Research Databases  Search Screen - Advanced Search  Database - CINAHL Plus with Full Text | 61,080 |
| --- | --- | --- | --- | --- |

|  | AMED |  |  |  |
| --- | --- | --- | --- | --- |
| **#** | **Query** | **Limiters/Expanders** | **Last Run Via** | **Results** |
| S69 | S7 AND S23 AND S67 AND S68 | Expanders - Apply equivalent subjects  Search modes - Find all my search terms | Interface - EBSCOhost Research Databases  Search Screen - Advanced Search Database - AMED - The Allied and Complementary Medicine Database | 1 |
| S68 | S8 OR S9 OR S10 OR S11 OR S12 OR S13 OR S14 OR S15 OR S16 OR S17 | Expanders - Apply equivalent subjects  Search modes - Find all my search terms | Interface - EBSCOhost Research Databases  Search Screen - Advanced Search Database - AMED - The Allied and Complementary Medicine Database | 41,113 |
| S67 | S24 OR S25 OR S26 OR S27 OR S28 OR S29 OR S30 OR S31 OR S32 OR S33 OR S34 OR S35 OR S36 OR S37 OR S38 OR S39 OR S40 OR S41 OR S42 OR S43 OR S44 OR S45 OR S46 OR S47 OR S48 OR S49 OR S50 OR S51 OR S52 OR S53 OR S54 OR S55 OR S56 OR S57 OR S58 OR S59 OR S60 OR S61 OR S62 OR S63 OR S64 OR S65 OR S66 | Expanders - Apply equivalent subjects  Search modes - Find all my search terms | Interface - EBSCOhost Research Databases  Search Screen - Advanced Search Database - AMED - The Allied and Complementary Medicine Database | 57,572 |
| S66 | ((herbal or Chinese or ayurved*) N/2 (therap* or medicine or treatment or intervention)) | Expanders - Apply equivalent subjects  Search modes - SmartText Searching | Interface - EBSCOhost Research Databases  Search Screen - Advanced Search Database - AMED - The Allied and Complementary Medicine Database | 2 |
| S65 | "medicinal herbs" or "medicinal teas" | Expanders - Apply equivalent subjects  Search modes - Find all my search terms | Interface - EBSCOhost Research Databases  Search Screen - Advanced Search Database - AMED - The Allied and Complementary Medicine Database | 317 |
| S64 | "diet therapy" or "nutrition intervention" | Expanders - Apply equivalent subjects  Search modes - Find all my search terms | Interface - EBSCOhost Research Databases  Search Screen - Advanced Search Database - AMED - The Allied and Complementary Medicine Database | 2,217 |
| S63 | "traditional medicine" or "complementary medicine" or "alternative medicine" or "herb medicine" | Expanders - Apply equivalent subjects  Search modes - Find all my search terms | Interface - EBSCOhost Research Databases  Search Screen - Advanced Search Database - AMED - The Allied and Complementary Medicine Database | 17,889 |

| S62 | "traditional chinese medicine" or tcm or "chinese medicine" | Expanders - Apply equivalent subjects  Search modes - Find all my search terms | Interface - EBSCOhost Research Databases  Search Screen - Advanced Search Database - AMED - The Allied and Complementary Medicine Database | 9,380 |
| --- | --- | --- | --- | --- |
| S61 | "Ayurvedic medicine" | Expanders - Apply equivalent subjects  Search modes - Find all my search terms | Interface - EBSCOhost Research Databases  Search Screen - Advanced Search Database - AMED - The Allied and Complementary Medicine Database | 1,397 |
| S60 | phytotherapy or "herbal medicine" | Expanders - Apply equivalent subjects  Search modes - Find all my search terms | Interface - EBSCOhost Research Databases  Search Screen - Advanced Search Database - AMED - The Allied and Complementary Medicine Database | 12,312 |
| S59 | Herb* or spice* | Expanders - Apply equivalent subjects  Search modes - Find all my search terms | Interface - EBSCOhost Research Databases  Search Screen - Advanced Search Database - AMED - The Allied and Complementary Medicine Database | 20,092 |
| S58 | Chandrashoor or "Lepidium sativum" | Expanders - Apply equivalent subjects  Search modes - Find all my search terms | Interface - EBSCOhost Research Databases  Search Screen - Advanced Search Database - AMED - The Allied and Complementary Medicine Database | 19 |
| S57 | "red clover" or "Trifolium pratense" | Expanders - Apply equivalent subjects  Search modes - Find all my search terms | Interface - EBSCOhost Research Databases  Search Screen - Advanced Search Database - AMED - The Allied and Complementary Medicine Database | 26 |
| S56 | "Blessed thistle" or "Cnicus benedictus" | Expanders - Apply equivalent subjects  Search modes - Find all my search terms | Interface - EBSCOhost Research Databases  Search Screen - Advanced Search Database - AMED - The Allied and Complementary Medicine Database | 4 |

| S55 | Alfalfa or "Medicago sativa" | Expanders - Apply equivalent subjects Search modes - Find all my search terms | Interface - E Search Scr  Database - Medicine D |
| --- | --- | --- | --- |

| S54 | dill or "Anethum graveolens" | Expanders - Apply equivalent subjects  Search modes - Find all my search terms | Interface - EBSCOhost Research Databases  Search Screen - Advanced Search Database - AMED - The Allied and Complementary Medicine Database | 39 |
| --- | --- | --- | --- | --- |
| S53 | coriander or cilantro or "Coriandrum sativum" | Expanders - Apply equivalent subjects  Search modes - Find all my search terms | Interface - EBSCOhost Research Databases  Search Screen - Advanced Search Database - AMED - The Allied and Complementary Medicine Database | 55 |
| S52 | blackseed or "Nigella sativa" | Expanders - Apply equivalent subjects  Search modes - Find all my search terms | Interface - EBSCOhost Research Databases  Search Screen - Advanced Search Database - AMED - The Allied and Complementary Medicine Database | 128 |
| S51 | ginger or "zingiber officinale" | Expanders - Apply equivalent subjects  Search modes - Find all my search terms | Interface - EBSCOhost Research Databases  Search Screen - Advanced Search Database - AMED - The Allied and Complementary Medicine Database | 365 |
| S50 | "banana flower" or "musa x paradisiaca" | Expanders - Apply equivalent subjects  Search modes - Find all my search terms | Interface - EBSCOhost Research Databases  Search Screen - Advanced Search Database - AMED - The Allied and Complementary Medicine Database | 2 |
| S49 | "lemon grass" or "cymbopogon citratus" | Expanders - Apply equivalent subjects  Search modes - Find all my search terms | Interface - EBSCOhost Research Databases  Search Screen - Advanced Search Database - AMED - The Allied and Complementary Medicine Database | 54 |
| S48 | anise or "pimpinella anisum" | Expanders - Apply equivalent subjects  Search modes - Find all my search terms | Interface - EBSCOhost Research Databases  Search Screen - Advanced Search Database - AMED - The Allied and Complementary Medicine Database | 59 |

| S47 | caraway or "carum carvi" | Expanders - Apply equivalent subjects  Search modes - Find all my search terms | Interface - EBSCOhost Research Databases  Search Screen - Advanced Search  Database - AMED - The Allied and Complementary Medicine  Database | 47 |
| --- | --- | --- | --- | --- |

| S46 | Melissa or "melisa officinalis" | Expanders - Apply equivalent subjects  Search modes - Find all my search terms | Interface - EBSCOhost Research Databases  Search Screen - Advanced Search  Database - AMED - The Allied and Complementary Medicine Database | 86 |
| --- | --- | --- | --- | --- |
| S45 | "stinging nettle" or "urtica diocia" | Expanders - Apply equivalent subjects  Search modes - Find all my search terms | Interface - EBSCOhost Research Databases  Search Screen - Advanced Search  Database - AMED - The Allied and Complementary Medicine Database | 21 |
| S44 | Carduus or "Cardus marianus" | Expanders - Apply equivalent subjects  Search modes - Find all my search terms | Interface - EBSCOhost Research Databases  Search Screen - Advanced Search  Database - AMED - The Allied and Complementary Medicine Database | 11 |
| S43 | Mulunggay or "moringa oleifera" | Expanders - Apply equivalent subjects  Search modes - Find all my search terms | Interface - EBSCOhost Research Databases  Search Screen - Advanced Search  Database - AMED - The Allied and Complementary Medicine Database | 88 |
| S42 | "Goat's rue" or "Galega officinalis" | Expanders - Apply equivalent subjects  Search modes - Find all my search terms | Interface - EBSCOhost Research Databases  Search Screen - Advanced Search  Database - AMED - The Allied and Complementary Medicine Database | 9 |
| S41 | Chasteberry or "vitex agnus castus" | Expanders - Apply equivalent subjects  Search modes - Find all my search terms | Interface - EBSCOhost Research Databases  Search Screen - Advanced Search  Database - AMED - The Allied and Complementary Medicine Database | 37 |
| S40 | "milk thistle" or "silybum marianum" or "silymarin phosphatidylserine" | Expanders - Apply equivalent subjects  Search modes - Find all my search terms | Interface - EBSCOhost Research Databases  Search Screen - Advanced Search  Database - AMED - The Allied and Complementary Medicine Database | 131 |
| S39 | fennel or "foeniculi vulgare mill" | Expanders - Apply equivalent subjects  Search modes - Find all my search terms | Interface - EBSCOhost Research Databases  Search Screen - Advanced Search  Database - AMED - The Allied and | 72 |

|  |  |  | Complementary Medicine Database |  |
| --- | --- | --- | --- | --- |
| S38 | Torbangun or "Coleus amboinicus lour" | Expanders - Apply equivalent subjects  Search modes - Find all my search terms | Interface - EBSCOhost Research Databases  Search Screen - Advanced Search  Database - AMED - The Allied and Complementary Medicine Database | 1 |
| S37 | "Shatavari" or "Asparagus racemosus" | Expanders - Apply equivalent subjects  Search modes - Find all my search terms | Interface - EBSCOhost Research Databases  Search Screen - Advanced Search  Database - AMED - The Allied and Complementary Medicine  Database | 29 |

| S36 | Damiana or "Turnera diffusa" | Expanders - Apply equivalent subjects  Search modes - Find all my search terms | Interface - EBSCOhost Research Databases  Search Screen - Advanced Search  Database - AMED - The Allied and Complementary Medicine Database | 12 |
| --- | --- | --- | --- | --- |
| S35 | "Raspberry leaf" or "Rubus idaeus" | Expanders - Apply equivalent subjects  Search modes - Find all my search terms | Interface - EBSCOhost Research Databases  Search Screen - Advanced Search  Database - AMED - The Allied and Complementary Medicine Database | 12 |
| S34 | Fenugreek or "Trigonella foenumgraecum" | Expanders - Apply equivalent subjects  Search modes - Find all my search terms | Interface - EBSCOhost Research Databases  Search Screen - Advanced Search  Database - AMED - The Allied and Complementary Medicine Database | 74 |
| S33 | herb* N/2 galact* | Expanders - Apply equivalent subjects  Search modes - SmartText Searching | Interface - EBSCOhost Research Databases  Search Screen - Advanced Search  Database - AMED - The Allied and Complementary Medicine Database | 334 |
| S32 | insufficie* N/2 milk* | Expanders - Apply equivalent subjects  Search modes - SmartText Searching | Interface - EBSCOhost Research Databases  Search Screen - Advanced Search | 63 |

|  |  |  | Database - AMED - The Allied and Complementary Medicine Database |  |
| --- | --- | --- | --- | --- |
| S31 | "enhanc* lactat*" | Expanders - Apply equivalent subjects  Search modes - Find all my search terms | Interface - EBSCOhost Research Databases  Search Screen - Advanced Search  Database - AMED - The Allied and Complementary Medicine Database | 1 |

| S30 | "stimula* lactat*" | Expanders - Apply equivalent subjects  Search modes - Find all my search terms | Interface - EBSCOhost Research Databases  Search Screen - Advanced Search Database - AMED - The Allied and Complementary Medicine Database | 1 |
| --- | --- | --- | --- | --- |
| S29 | "promot* lactat*" | Expanders - Apply equivalent subjects  Search modes - Find all my search terms | Interface - EBSCOhost Research Databases  Search Screen - Advanced Search Database - AMED - The Allied and Complementary Medicine Database | 4 |
| S28 | lactogenic | Expanders - Apply equivalent subjects  Search modes - Find all my search terms | Interface - EBSCOhost Research Databases  Search Screen - Advanced Search Database - AMED - The Allied and Complementary Medicine Database | 3 |
| S27 | "plant extract*" | Expanders - Apply equivalent subjects  Search modes - Find all my search terms | Interface - EBSCOhost Research Databases  Search Screen - Advanced Search Database - AMED - The Allied and Complementary Medicine Database | 24,911 |
| S26 | "medicinal plant*" | Expanders - Apply equivalent subjects  Search modes - Find all my search terms | Interface - EBSCOhost Research Databases  Search Screen - Advanced Search Database - AMED - The Allied and Complementary Medicine Database | 3,796 |
| S25 | galactagogue | Expanders - Apply equivalent subjects  Search modes - Find all my search terms | Interface - EBSCOhost Research Databases  Search Screen - Advanced Search Database - AMED - The Allied and  Complementary Medicine Database | 12 |

| S24 | galactogogue | Expanders - Apply equivalent subjects  Search modes - Find all my search terms | Interface - EBSCOhost Research Databases  Search Screen - Advanced Search Database - AMED - The Allied and Complementary Medicine Database | 5 |
| --- | --- | --- | --- | --- |
| S23 | S18 OR S19 OR S20 OR S21 OR S22 | Expanders - Apply equivalent subjects  Search modes - Find all my search terms | Interface - EBSCOhost Research Databases  Search Screen - Advanced Search Database - AMED - The Allied and Complementary Medicine Database | 30,674 |
| S22 | "clinical trial*" or "control* clinical trial*" | Expanders - Apply equivalent subjects  Search modes - Find all my search terms | Interface - EBSCOhost Research Databases  Search Screen - Advanced Search Database - AMED - The Allied and Complementary Medicine Database | 7,658 |
| S21 | random* | Expanders - Apply equivalent subjects  Search modes - Find all my search terms | Interface - EBSCOhost Research Databases  Search Screen - Advanced Search Database - AMED - The Allied and Complementary Medicine Database | 25,880 |
| S20 | placebo* | Expanders - Apply equivalent subjects  Search modes - Find all my search terms | Interface - EBSCOhost Research Databases  Search Screen - Advanced Search Database - AMED - The Allied and Complementary Medicine Database | 4,187 |
| S19 | random* or placebo* or "single blind*" or "double blind*" or "triple blind*" | Expanders - Apply equivalent subjects  Search modes - Find all my search terms | Interface - EBSCOhost Research Databases  Search Screen - Advanced Search Database - AMED - The Allied and Complementary Medicine Database | 27,595 |
| S18 | "randomized control* trial*" or rtc or "randomised control* trial*" | Expanders - Apply equivalent subjects  Search modes - Find all my search terms | Interface - EBSCOhost Research Databases  Search Screen - Advanced Search Database - AMED - The Allied and Complementary Medicine Database | 10,191 |

| S17 | (milk* N/2 (suppl* or volume* or quantity or  yield*)) | Expanders - Apply equivalent subjects  Search modes - SmartText Searching | Interface - EBSCOhost Research Databases  Search Screen - Advanced Search  Database - AMED - The Allied and Complementary Medicine  Database | 86 |
| --- | --- | --- | --- | --- |

| S16 | express* or extract* | Expanders - Apply equivalent subjects  Search modes - Find all my search terms | Interface - EBSCOhost Research Databases  Search Screen - Advanced Search  Database - AMED - The Allied and Complementary Medicine Database | 40,060 |
| --- | --- | --- | --- | --- |
| S15 | hand* N/2 express* | Expanders - Apply equivalent subjects  Search modes - SmartText Searching | Interface - EBSCOhost Research Databases  Search Screen - Advanced Search  Database - AMED - The Allied and Complementary Medicine Database | 74 |
| S14 | "breast pump*" or breastpump* or "breast pumping" | Expanders - Apply equivalent subjects  Search modes - SmartText Searching | Interface - EBSCOhost Research Databases  Search Screen - Advanced Search  Database - AMED - The Allied and Complementary Medicine Database | 7 |
| S13 | ((milk* or breast*) N/2 express*) | Expanders - Apply equivalent subjects  Search modes - SmartText Searching | Interface - EBSCOhost Research Databases  Search Screen - Advanced Search  Database - AMED - The Allied and Complementary Medicine Database | 122 |
| S12 | ((breast* or mother* or human or maternal*) N/2 milk) | Expanders - Apply equivalent subjects  Search modes - SmartText Searching | Interface - EBSCOhost Research Databases  Search Screen - Advanced Search  Database - AMED - The Allied and Complementary Medicine Database | 176 |

| S11 | "human milk" or "breast milk" or breastmilk | Expanders - Apply equivalent subjects  Search modes - Find all my search terms | Interface - EBSCOhost Research Databases  Search Screen - Advanced Search  Database - AMED - The Allied and  Complementary Medicine Database | 51 |
| --- | --- | --- | --- | --- |

| S10 | lactat* | Expanders - Apply equivalent subjects  Search modes - Find all my search terms | Interface - EBSCOhost Research  Databases  Search Screen - Advanced Search Database - AMED - The Allied and Complementary Medicine Database | 1,230 |
| --- | --- | --- | --- | --- |
| S9 | (breast* N/2 (fed* or feed*)) | Expanders - Apply equivalent subjects  Search modes - SmartText Searching | Interface - EBSCOhost Research Databases  Search Screen - Advanced Search Database - AMED - The Allied and Complementary Medicine Database | 78 |
| S8 | breastfeed* or "breast feed*" | Expanders - Apply equivalent subjects  Search modes - Find all my search terms | Interface - EBSCOhost Research Databases  Search Screen - Advanced Search Database - AMED - The Allied and Complementary Medicine Database | 156 |
| S7 | S1 OR S2 OR S3 OR S4 OR S5 OR S6 | Expanders - Apply equivalent subjects  Search modes - Find all my search terms | Interface - EBSCOhost Research Databases  Search Screen - Advanced Search Database - AMED - The Allied and Complementary Medicine Database | 664 |
| S6 | TI ( (nursing N2 (maternal* or mother* or baby or babies or infant* or newborn* or neonat* or "neo nat*" or perinat* or "peri nat*" or premie or premies)) ) OR AB ( (nursing N2 (maternal* or mother* or baby or babies or infant* or newborn* or neonat* or "neo nat*" or perinat* or "peri nat*" or premie or premies)) ) | Expanders - Apply equivalent subjects  Search modes - SmartText Searching | Interface - EBSCOhost Research Databases  Search Screen - Advanced Search Database - AMED - The Allied and Complementary Medicine Database | 308 |
| S5 | "very low birth weight" or "vlbw" | Expanders - Apply equivalent subjects  Search modes - Find all my search terms | Interface - EBSCOhost Research Databases  Search Screen - Advanced Search Database - AMED - The Allied and Complementary Medicine Database | 42 |
| S4 | ("low birth weight" or "lbw") | Expanders - Apply equivalent subjects  Search modes - Find all my search terms | Interface - EBSCOhost Research Databases  Search Screen - Advanced Search Database - AMED - The Allied and Complementary Medicine Database | 147 |
| S3 | (neonatal N/3 unit*) | Expanders - Apply equivalent subjects  Search modes - SmartText Searching | Interface - EBSCOhost Research Databases  Search Screen - Advanced Search Database - AMED - The Allied and  Complementary Medicine Database | 193 |

| S2 | nicu or "neonatal intensive care unit*" or "special care" or "baby unit*" or "newborn intensive care" | Expanders - Apply equivalent subjects  Search modes - Find all my search terms | Interface - EBSCOhost Research Databases  Search Screen - Advanced Search Database - AMED - The Allied and Complementary Medicine Database | 230 |
| --- | --- | --- | --- | --- |

| S1 | (Premature or "pre-mature" or pretrem or "pre-term") N2  (baby or birth or neonat* or babies or infant*) | Expanders - Apply equivalent subjects  Search modes - Find all my search terms | Interface - EBSCOhost Research Databases  Search Screen - Advanced Search Database - AMED - The Allied and  Complementary Medicine Database | 326 |
| --- | --- | --- | --- | --- |

## Search Strategy from ProQuest

August 19 2024 07:52

| Set No. | Searched for | Databases | Results |
| --- | --- | --- | --- |
| S1 | noft(lactation OR breast feeding OR breast milk) AND noft(herbal) | ProQuest Dissertations & Theses Global | 19 |
