# Supplementary material for: Herbal galactagogues to improve breastmilk production and lactation in mothers of preterm babies: a systematic review of clinical trials
Source: Eur J Clin Nutr. 2025 Dec 5;80(2):146–58. doi: 10.1038/s41430-025-01679-x (PMC12929060; doi:10.1038/s41430-025-01679-x)
Supplement: Supplementary file 3 — Supplementary Materials 3 [file 41430_2025_1679_MOESM3_ESM.pdf]

Supplementary Figures 1 and 2

Supplementary figure 1: Milk volumes day 1

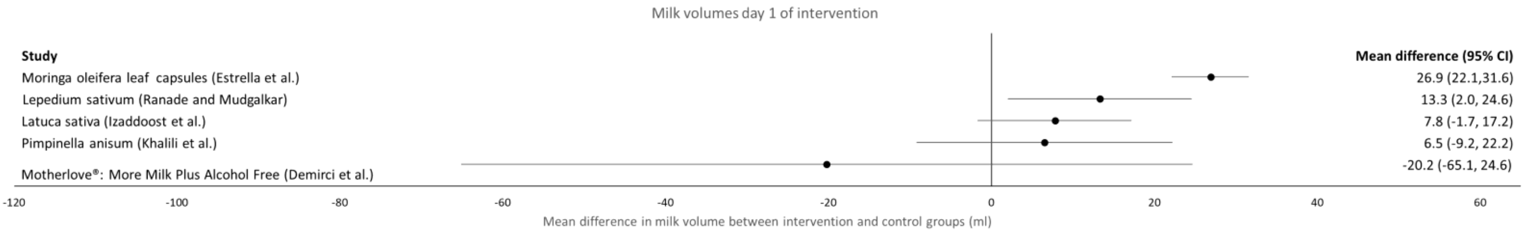

Supplementary figure 2: Milk volumes day 4

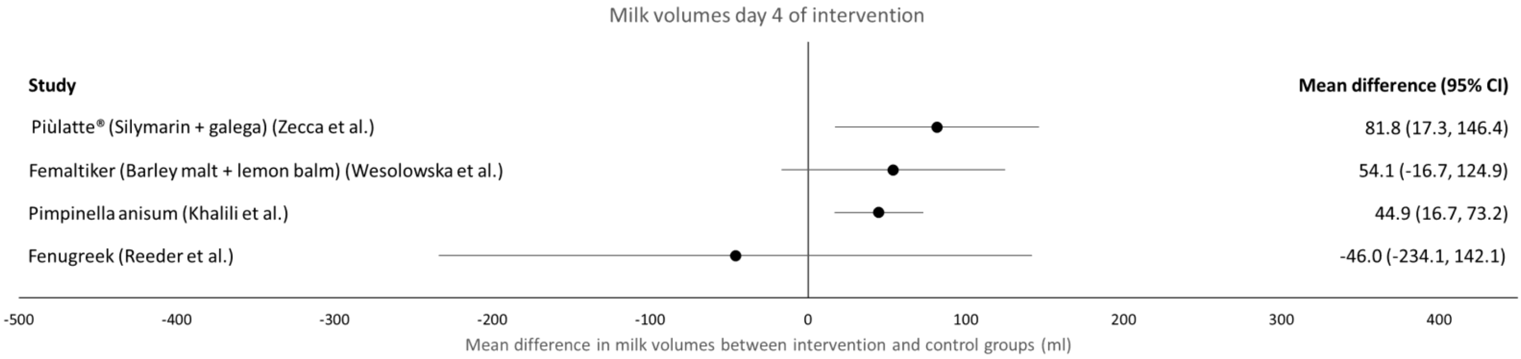

Supplementary tables 3-8

Supplementary table 3: Milk volumes day 1 of intervention

Study	Intervention	Intervention group mean milk volume (mls) (95% CI's)	Control group 1* mean milk volume (ml) (95% CI's)	Control group 2** mean milk volume (ml) (95% CI's)	Mean difference between intervention and control group 1 (95% CI's)
Estrella et al, 2000	Moringa oleifera lea capsules 250mg	114.1 (91.0 to 137.2)	87.2 (70.8 to 103.6)	N/A	26.9 (22.1 to 31.6)
Ranade and Mudgalkar 2021	*Lepidium sativum*	66.3 (56.5 to 76.1)	53.0 (46.8 to 59.3)	N/A	13.3 (2.0 to 24.6)
Khalili et al. 2023	*Pimpinella anisum* herbal tea	137.9 (126.0 to 149.8)	131.4 (121.7 to 141.1)	112.8 (97.5 to 128.1)	6.5 (-9.2 to 22.2)
Izaddoost et al. 2023	*Lactuca sativa* syrup	141.7 (134.4 to 149.0)	133.9 (127.8 to 140.1)	134.9 (130.6 to 139.2)	7.8 (-1.7 to 17.2)

* Estrella et al's control group 1 received matching placebo capsules. Demirci et al.'s control group 1 were given a meditation intervention. Ranade and Mudgalkar's control group 1 were given no intervention. Khalili et al.'s control group 1 was given a placebo of black tea. Izaddoost et al.'s control group 1 was given a placebo of sugar syrup.

** Demirci et al. did not have a control group 2. Ozalkaya et al.'s control group 2 was given advice on supportive measures. Ranade and Mudgalkar did not have a control group 2. Khalili et al.'s control group 2 was given no treatment. Izaddoost et al.'s control group 2 was given no intervention

N/A = not applicable; NI = No information provided; CI = confidence interval

Supplementary table 4: Milk volumes day 4 of intervention

Study	Intervention	Intervention group mean milk volume (ml) (95% CI's)	Control group 1* mean milk volume (ml) (95% CI's)	Control group 2** mean milk volume (ml) (95% CI's)	Mean difference between intervention and control group 1 (95% CI's)
Reeder et al. 2013***	Fenugreek	345 (230.7 to 459.3)	391 (211.8 to 570.2)	N/A	-46 (142.1 to -234.1)
Zecca et al. 2016	Piùlatte®	215.8 (160.1 to 271.5)	134.0 (100.1 to 167.8)	N/A	81.8 (17.3 to 146.4)
Wesolowska et al. 2021	Femaltiker®	203.4 (149.4 to 257.4)	149.3 (101.8 to 196.8)	N/A	54.1 (-16.7 to 124.9)
Khalili et al. 2023	*Pimpinella anisum* herbal tea	193.7 (170.0 to 217.4)	148.8 (132.6 to 165.0)	130.2 (110.5 to 149.9)	44.9 (16.7 to 73.2)
Izaddoost et al. 2023	*Latuca sativa* syrup	198.3 (183.3 to 213.4)	159.1 (106.2 to 212.1)	152.8 (145.4 to 160.2)	39.2 (22.4 to 56.0)

*Reeder et al.'s control group 1 were given a placebo of starch capsules. Zecca et al.'s control group 1 were given a placebo of lactose. Wesolowska et al.'s control group 1 were given a placebo blend of sucrose, apple fiber and natural aroma caramel. Khalili et al.'s control group 1 was given a placebo of black tea. Izaddoost et al.'s control group 1 given a placebo of sugar syrup

** Khalili et al.'s control group 2 was given no treatment. Izaddoost et al.'s control group 2 given no treatment. No other included studies for day 5 had a second control group.

***Day 4 results not provided; day 5 results used for comparison

N/A = not applicable; NI = No information provided; CI = confidence interval

Supplementary table 5: Milk volumes day 7 of intervention

Study	Intervention	Intervention group mean milk volume (ml) (95% CI's)	Control group 1* milk mean volume (ml) (95% CI's)	Control group 2** mean milk volume (ml) (95% CI's)	Mean difference between intervention and control group 1 (95% CI's)
Reeder et al. 2013***	Fenugreek	514<br>(311.4 to 716.6)	577<br>322.9 to 831.2	N/A	-63.0ml<br>(-354.6 to 228.6)
Peila et al. 2015	BIO-C®	420.0 (277.0 to 563.0)	350.0 (239.0 to 247.0)	N/A	70.0 (-57.8 to 197.8)
Zecca et al. 2016	Piùlatte®	268.4<br>(203.0 to 333.8)	163.4<br>(117.5 to 209.4)	N/A	104.98<br>(27.1 to 182.9)
Demirci et al. 2016****	Motherlove: More Milk Plus Alcohol Free ®	52.6<br>(21.2 to 83.9)	29.2<br>(-23.2 to 81.7)	N/A	23.3<br>(-15.8 to 62.5)
Ozalkaya et al. 2018	Hipp Natal® herbal tea mixture containing 1.0% *Urtica dioica*	577.5<br>(NI)	477.7<br>(NI)	422.6<br>(NI)	99.8<br>(Not possible to calculate)
Ranade and Mudgalkar 2021	*Lepidium sativum*	123.2<br>(114.4 to 132.0)	123.4<br>(115.8 to 131.0)	N/A	-0.2<br>(-11.0 to 10.6)
Wesolowska et al. 2021	Femaltiker®	508.6<br>(411.0 to 606.2)	359.5<br>(299.4 to 419.6)	N/A	149.1<br>(38.1 to 260.1)
Khalili et al. 2023	*Pimpinella anisum* herbal tea	268.1<br>(238.5 to 297.7)	170.1<br>(150.1 to 190.1)	154.8<br>(128.1-181.4)	98.0<br>(63.2 to 132.8)
Izaddoost et al. 2023	*Lactuca sativa* syrup	266.8 (246.8 to 286.8)	184.8 (174.1 to 195.5)	176.0 (164.4 to 187.5)	82.0 (59.5 to 104.5)

*Reeder et al.'s control group 1 were given a placebo of starch capsules. Peila et al.'s control group 1 were given a placebo of maltodextrin. Zecca et al.'s control group 1 were given a placebo of lactose. Demirci et al.'s control group 1 were given a meditation intervention. Ozalkaya et al.'s control group 1 were given a placebo of fruit tea. Ranade and Mudgalkar's control group 1 were given no intervention. Wesolowska et al.'s control group 1 were given a placebo blend of sucrose, apple fiber and natural aroma caramel. Khalili et al.'s control group 1 was given a placebo of black tea. Izaddoost et al.'s control group 1 given a placebo of sugar syrup

**Ozalkaya et al.'s control group 2 was given advice on supportive measures. Khalili et al.'s control group 2 was given no intervention. Izaddoost et al.'s control group 2 were given no intervention.

***Day 7 values not provided, so day 10 values used for comparison

****This study used a meditation intervention as a comparator. Participants pumped 2 times per day after direct breastfeeding. If not directly breastfeeding, they recorded 2 volumes from a pump at 2 time points in a day. We used the values from pump 2.

Supplementary table 6: Milk volumes day 28 of intervention

Study	Intervention	Intervention group mean total milk volume for study duration (ml) (95% CI's)	Control group 1* mean total milk volume for study duration (ml) (95% CI's)	Mean difference between intervention and control group 1 (95% CI's)
Peila et al. 2015	BIO-C	376.8 (216.8 to 536.8)	345.6 (223.4 to 467.8)	31.2 (-165.1 to 227.5)
Ranade and Mudgalkar 2021	*Lepidium sativum*	552.6 (492.9 to 612.3)	485.4 (441.4 to 529.4)	67.2ml (-2.9 to 137.3)

*Peila et al.'s control group 1 were given a placebo of maltodextrin. Ranade and Mudgalkar's control group 1 were given no intervention.

N/A = not applicable NI = No information provided CI = confidence interval

Supplementary table 7: Total milk volumes over intervention duration

Study	Intervention	Intervention group mean total milk volume for study duration (ml) (95% CI's)	Control group 1* mean total milk volume for study duration (ml) (95% CI's)	Mean difference between intervention and control group 1 (95% CI's)
Zecca et al. 2016	Piùlatte®	6523 (5017.0 to 8030.4)	4136.0 (2972.5 to 5299.5)	2387.0 (531.3 to 4242.7)
Wesolowska et al. 2021	Femaltiker®	6036.0 (5028.5 to 7043.5)	4209.0 (3531.3 to 4886.7)	1827.0 (650.7 to 3003.4)

*Zecca et al.'s control group 1 were given a placebo of lactose. Wesolowska et al.'s control group 1 were given a placebo blend of sucrose, apple fiber and natural aroma caramel.

N/A = not applicable NI = No information provided CI = confidence interval

Supplementary table 8: Infant weight day 7 of intervention

Study	Intervention	Intervention group mean infant weight (g) (95% CI's)	Control group 1* mean infant weight (g) (95% CI's)	Control group 2** mean infant weight (g) (95% CI's)	Mean difference between intervention and control group 1 (95% CI's)
Demirci et al. 2016***	Motherlove: More Milk Plus Alcohol Free ®	3252.5 (2634.8 to 3870.2)	3476.4 (2589.4 to 4363.4)	N/A	-223.9 (-879.0 to 431.2)
Ozalkaya et al. 2018*****	Hipp Natal® Herbal tea mixture containing 1.0% stinging nettle (*Urtica dioica*)	1550.0 (NI)	1553.0 (NI)	1551.0 (NI)	-3.0 (Not able to be calculated)
Khalili et al. 2023*****	*Pimpinella anisum* herbal tea	1370.1 (1319.4 to 1420.8)	1360.9 (1317.4 to 1404.4)	1321.3 (1268.8 to 1373.8)	9.2 (-55.8 to 74.2)
Izaddoost et al. 2023****	*Latuca sativa* syrup	68.1 (59.3 to 76.8)	95.3 (52.1 to 138.4)	99.1 (90.6 to 107.5)	-27.2 (-74.6 to 20.2)

*Demirci et al.'s control group 1 were given a meditation intervention. Ozalkaya et al.'s control group 1 were given a placebo of fruit tea. Khalili et al.'s control group 1 was given a placebo of black tea. Izaddoost et al.'s control group 1 given a placebo of sugar syrup

**Ozalkaya et al.'s control group 2 was given advice on supportive measures. Khalili et al.'s control group 2 was given no intervention. Izaddoost et al.'s control group 2 was given no intervention.

***This study used a meditation intervention as a comparator.

****This study provided increase in infant weight values rather than raw infant weights

*****These studies allowed infants to be fed formula in addition to breastmilk

N/A = not applicable NI = No information provided CI = confidence interval
